# Microbial community structure in hadal sediments: high similarity along trench axes and strong changes along redox gradients

**DOI:** 10.1038/s41396-021-01021-w

**Published:** 2021-06-08

**Authors:** Clemens Schauberger, Ronnie N. Glud, Bela Hausmann, Blandine Trouche, Lois Maignien, Julie Poulain, Patrick Wincker, Sophie Arnaud-Haond, Frank Wenzhöfer, Bo Thamdrup

**Affiliations:** 1 Hadal & Nordcee, Department of Biology, University of Southern Denmark, Odense, Denmark; 2 Department of Ocean and Environmental Sciences, Tokyo University of Marine Science and Technology, Tokyo, Japan; 3 Danish Institute of Advanced Study (DIAS), University of Southern Denmark, Odense, Denmark; 4 Joint Microbiome Facility of the Medical University of Vienna and the University of Vienna, Vienna, Austria; 5 Department of Laboratory Medicine, Medical University of Vienna, Vienna, Austria; 6 Univ Brest, CNRS, IFREMER, Microbiology of Extreme Environments Laboratory, Plouzané, France; 7 Génomique Métabolique, Genoscope, Institut François Jacob, CEA, CNRS, Univ Evry, Université Paris-Saclay, Evry, France; 8 MARBEC, Institut Français de Recherche pour L’Exploitation de la Mer, Univ Montpellier, CNRS, IRD, Sète, France; 9 Alfred Wegener Institute, Helmholtz Center for Polar and Marine Research, Bremerhaven, Germany; 10 Max Planck Institute for Marine Microbiology and Ecology, Bremen, Germany

**Keywords:** Water microbiology, Microbial ecology, Microbiome, Biogeochemistry, Biogeochemistry

## Abstract

Hadal trench sediments are hotspots of biogeochemical activity in the deep sea, but the biogeochemical and ecological factors that shape benthic hadal microbial communities remain unknown. Here, we sampled ten hadal sites from two trench regions with a vertical resolution of down to 1 cm. We sequenced 16S rRNA gene amplicons using universal and archaea-specific primer sets and compared the results to biogeochemical parameters. Despite bathymetric and depositional heterogeneity we found a high similarity of microbial communities within each of the two trench axes, while composition at the phylum level varied strongly with sediment depth in conjunction with the redox stratification into oxic, nitrogenous, and ferruginous zones. As a result, communities of a given sediment horizon were more similar to each other across a distance of hundreds of kilometers within each trench, than to those of adjacent horizons from the same sites separated only by centimeters. Total organic carbon content statistically only explained a small part of the variation within and between trenches, and did not explain the community differences observed between the hadal and adjacent shallower sites. Anaerobic taxa increased in abundance at the top of the ferruginous zone, seeded by organisms deposited at the sediment surface and surviving burial through the upper redox zones. While an influence of other potential factors such as geographic isolation, hydrostatic pressure, and non-steady state depositional regimes could not be discerned, redox stratification and diagenesis appear to be the main selective forces that structure community composition in hadal sediments.

## Introduction

Marine sediments constitute one of the largest sinks of organic matter on the planet [1, 2] and benthic, microbially mediated processes contribute substantially to element cycling on a global scale [3, 4]. Benthic microbial communities, even in surface sediments, are dissimilar to their planktonic counterparts, their composition being driven by forces within the benthic environment [5]. In abyssal and bathyal surface sediments, a large fraction of the variation in community composition has been found to correlate with total organic carbon (TOC) content, yet the similarity between sites with similar TOC levels decreases with geographic distance, which suggests some degree of endemism at individual locations [6], while many benthic microbes still are cosmopolitans [7].

Sediments impose such severe constraints on microbial dispersal that the community in the surface layer must serve as seed stock for communities in deeper sediment horizons [8]. Indeed, recent studies concluded that selective survival is the predominant process defining the community structure in subsurface sediments, while evolutionary diversification could only play a very limited role [9–11]. During burial, benthic microbial communities experience a vertical biogeochemical zonation that results from interactions between microbially mediated and physical processes [12]. These gradients in turn must have a reciprocal selective effect on community composition with increasing sediment depth [13]. The successive depletion of different electron acceptors may be a strong selective force in particular [14]. Yet, we lack a detailed understanding of how seed stock community composition and biogeochemical factors interact to shape the succession of microbial communities across the metabolic gradient between oxic, nitrogenous, and ferruginous zones in marine sediments (definitions from Canfield and Thamdrup [15]).

As the depositional flux of organic matter is typically attenuated with increasing water depth [16], hadal sediments at depths of 6–11 km could be expected to represent the most organically depleted sediments in the oceans. By contrast, recent studies have found that hadal sediments generally have higher respiratory activities than adjacent abyssal settings located several kilometers closer to the surface [17, 18]. This is attributed to the funneling of organic carbon from the overlying water and to mass deposition by submarine landslides, which may bury labile organic carbon into deeper sediment sections [19, 20]. The dynamic depositional regime leads to horizontal and vertical heterogeneity [21], as reflected for instance in irregular depth distributions of organic carbon and microbial abundances [22]. The organic enrichment further results in a compression of the redox zonation relative to other deep-sea settings, and oxic, nitrogenous, and ferruginous zones develop in the upper decimeters of some hadal sediments [17, 23, 24]. This contrasts with adjacent abyssal sites, where carbon oxidation is almost entirely coupled to oxygen respiration, and oxygen penetration is typically on the order of decimeters [18].

These geophysical and biogeochemical characteristics imply that hadal sediments are unique benthic environments that cannot be understood simply through extrapolation of results from shallower water depths. Moreover, a better understanding the dynamics of hadal ecosystems may contribute to answering general questions related to the biogeography and assembly of microbial communities. Recent studies have shown that trenches harbor microbial communities that are notably dissimilar to those of adjacent abyssal settings [24, 25]. While communities also differed between trenches, the abundant taxa were not trench specific, indicating that differences between trenches were not driven by endemism [25]. Other factors such as variable TOC content and geophysical parameters were hypothesized to cause this distinction [24, 25], yet testing of this hypothesis, as well as investigation of the potential influence of other biogeochemical factors, is still lacking.

Here we explore the variability of microbial communities in hadal sediments along and between the axes of the Kermadec and Atacama trenches and relate community composition to redox stratification across oxic, nitrogenous, and ferruginous zones. We sequenced universal and archaea-specific 16S rRNA gene amplicons from over 450 sediment samples and compared the results to detailed, parallel biogeochemical investigations. We further compare the communities of the two trenches to adjacent off-axes sites at abyssal and bathyal depths and discuss drivers for the resolved dissimilarities.

## Material and methods

### Sample collection and DNA extraction

**Fig. 1:**
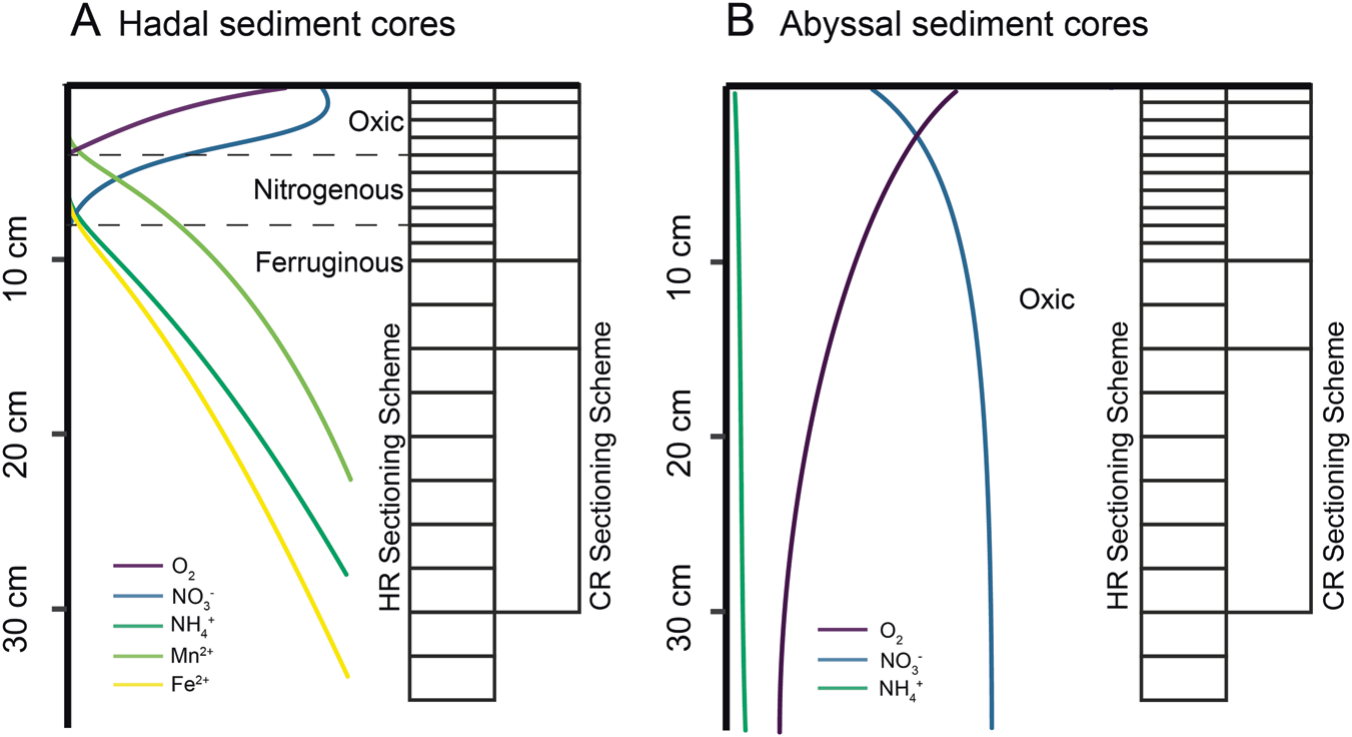
Redox stratification and sampling scheme. Schematic illustration of the redox stratification and associated porewater chemistry that were typical for hadal (**A**) and abyssal plain sediments (**B**) of this study. The stippled lines mark the borders of the individual redox zones. The grid of boxes to the right of each panel shows the coverage of the two core-sectioning schemes. HR, high resolution. CR, coarse resolution.

In the Kermadec Trench, sediment was collected at four locations (K3–K6) along the trench axis at 9300–10,010 m depth covering a distance of ~200 km, and one adjacent abyssal site (K7; 6080 m depth) on the subducting plate (Supplementary Table 1). Sampling in the Atacama Trench included six trench sites (A2–A6 and A10, 7720–8085 m depth, ~430 km distance), as well as two sites on the continental slope (A9 and A1, 4050 and 2560 m depth, respectively) and one abyssal site on the oceanic plate (A7, 5500 m depth). Sediment cores of 9.4 cm inner diameter were sampled by multicorer in the Atacama Trench and by multicorer (K6), boxcorer (K3, K4, K5, K7), and sediment lander (K4) in the Kermadec Trench. While at sites K3, K4, and K5, the turbidity of the overlying water in the boxcorer and the porewater chemistry indicated losses of the sediment surfaces (≤1, ≤1, and 5–7 cm, respectively), the sample of site K7 showed no sign of disturbance (unpublished data). The cores were sectioned upon shipboard arrival in a 3 °C cold room, using bleach and ethanol sterilized equipment. We processed three sediment cores at each site in the Atacama Trench: (i) two sediment cores were sectioned using a coarse-resolution (CR) scheme in 0–1, 1–3, 3–5, 5–10, 10–15 and 15–30 cm and 30–bottom (~35 cm) horizons, and (ii) one using both the CR scheme and a high-resolution (HR) scheme with sections of 1 cm at 0–10 cm sediment depth and 2.5 cm to the end of one core (~35 cm) (see also Fig. 1). In the Kermadec Trench we only used the CR sectioning scheme, as a response to the less steep biogeochemical gradient. Samples were frozen at –80 °C until DNA extraction in the laboratory, using the DNeasy PowerLyzer PowerSoil Kit [Qiagen] for the HR horizons, and the PowerMax Soil Kit [Qiagen] for the CR horizons. For details see the Supplementary Material and Methods section.

### Biogeochemical redox zone classification

We classified the sediment horizons as either oxic, nitrogenous, or ferruginous zones (see Fig. 1, Supplementary Table 1, and Supplementary Fig. 1) according to Canfield and Thamdrup [15] based on in situ oxygen profiles from the same locations [26], and the penetration depth of nitrate measured in parallel on board (Supplementary Table 1). Both the oxygen and nitrate profiles can be found in the PANGAEA repository under the project title “HADES-ERC” (www.pangaea.de). For further description on the procedures see the Supplementary Material and Methods section.

### Amplification and sequencing

Amplicons for barcoded sequencing on the HiSeq 2500 platform (2 × 250 paired-end) were produced using universal primers covering the V4 to V5 region of the 16S rRNA gene (515F-Y and 926R) [27] that were designed for marine samples and previously used by Peoples et al. (2019) in the Kermadec and Mariana trenches. Archaea-specific amplicons were generated using 517F and 958R [28] and sequenced with the same methodology. Unless otherwise mentioned, we report results based on the universal 16S rRNA gene dataset. For details see Brandt et al. [29] and the Supplementary Material and Methods section.

### Sequence processing and statistical analyses

The sequencing data were processed for each run independently using the Dada2 pipeline, and resulting amplicon sequence variants (ASVs) tables were then merged [30]. Negative controls were taken and treated equally to the samples along the entire laboratory process and used to determine putative contaminants. Contaminating ASVs were identified and filtered from the samples using the prevalence method of the Decontam R package [31]. Data management, visualization, and statistical analyses were done in the R studio environment, using the packages ampvis2 [32], phyloseq [33], vegan [34], ggplot2 [35], and dplyr [36]. We rarefied our data to equal sampling depths for visualizing the heatmaps, core microbiomes, and ASV frequency plots, whereas cumulative sum scaling normalization was applied using the metagenomeSeq R package [37] for Bray Curtis dissimilarity-based analyses, redundancy analysis, and variation partitioning. See the Supplementary Material and Methods section for details. The sequencing data can be accessed over the European nucleotide Archive under the project number PRJEB33873.

## Results and discussion

We successfully sequenced 16S rRNA gene amplicons from 454 samples with universal primers and 283 samples with archaea-specific primers, respectively (see Supplementary Table 2), and recovered 260,266 ASVs in the universal 16S rRNA gene dataset and 28,123 ASVs in the archaea-specific dataset (Supplementary Fig. 2). As samples from the Atacama Trench region included sectioning at higher depth resolution (HR sectioning scheme), we will present and discuss these first.

### Variability of microbial community composition along the Atacama Trench axis

The sediments of the Atacama Trench showed the shallowest oxygen penetrations found in any hadal trench to date, ranging from 4.1 cm at the southernmost site A6 to 3.1 cm at northernmost A10, and reflected a high organic carbon flux from the Humboldt upwelling system [22, 26]. Correspondingly, nitrate penetration depths ranged from 8 to only 6 cm with dissolved, ferrous iron accumulating below, while hydrogen sulfide was not detected (see Supplementary Fig. 1 and Supplementary Table 1).

**Fig. 2:**
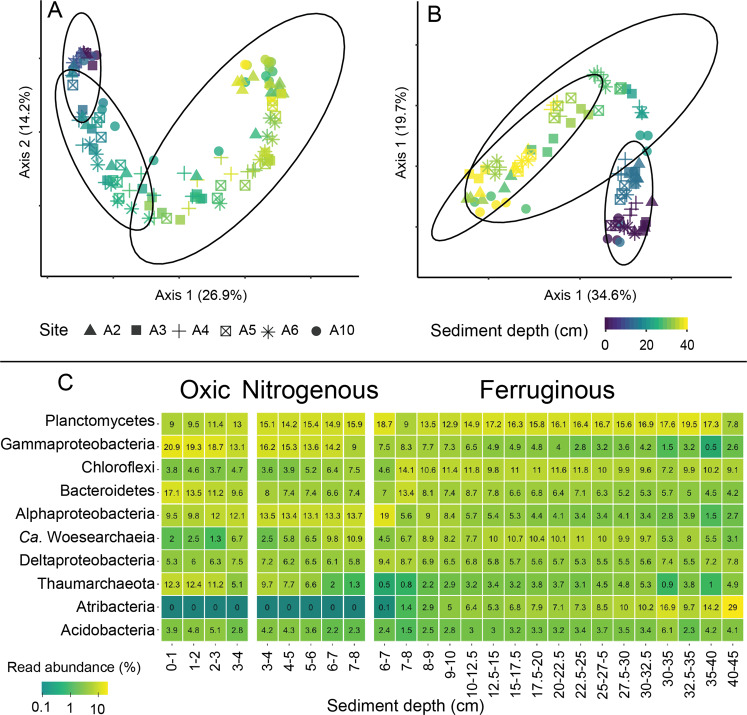
Microbial community composition in the Atacama Trench. Principal coordinate analysis (PCoA) of Bray Curtis dissimilarities between hadal samples from the Atacama Trench in the universal 16S rRNA gene (**A**) and archaea-specific 16S rRNA gene (**B**) data. The color gradient represents sediment depth and ovals mark the 95% confidence intervals of multivariate normal distributions of the oxic, nitrogenous, and ferruginous zones, respectively. Different symbols correspond to different sites. **C** Relative read abundance (%) on phylum/class level of the ten most abundant taxonomic groups (universal 16S rRNA gene data) grouped by redox zone and by depth within each zone in hadal samples from the Atacama Trench. Both the color gradient and the number within the squares indicate of the average relative read abundances within the respective sample group.

Comparison of the community structure obtained with universal primers within the Atacama Trench indicated very similar trends with sediment depth for all sites, with a gradual change from the sediment surface to deeper sediment horizons, and with only marginal overlap between individual redox zones (Fig. 2A). Similar patterns were observed using different dissimilarity metrics (Bray Curtis, weighted/unweighted UniFrac), ordination techniques (NMDS/t-SNE), and sectioning schemes (HR/CR; data not shown). The downcore gradient of microbial communities was also evident within individual redox zones. Thus, samples from the same sediment horizon (e.g., 1–2 cm) but from different sites, with geographic distances of up to 430 km, were more similar to each other than to their respective adjacent horizons, above (0–1 cm) or below (2–3 cm) (Supplementary Fig. 3A, B). The horizontal similarity was particularly pronounced in the upper part of the oxic zone, while it decreased toward the bottom of the nitrogenous zone and increased again in the ferruginous zone (Supplementary Fig. 3A, B). The CR sample subset contained triplicates from separate sediment cores originating from two multicorer deployments, and thus included two cores sampled within a distance of 0.1–1 m and one sampled at an estimated distance of 10–100 m from the other two. Triplicate samples from the same sediment horizon were more similar to each other (1 – Bray Curtis dissimilarity ~0.7–0.9) than to samples from the same horizons at other sites (~0.4–0.8; Supplementary Fig. 4A, C). This implies some increase of variability with geographic distance along the trench axis.

The steep change in community composition with increasing sediment depth yet relatively high similarity of communities from the same sediment depth at different sites was unexpected. Indeed, we expected that the irregular depositional regime of hadal trenches [18, 21], which was indicated in all hadal sediment cores through color layering and by site-specific fluctuations in depth distributions of porosity, TOC content, and cell numbers [22], would leave an imprint in the community. A closer examination of depth trends at individual sites based on Bray Curtis dissimilarity occasionally revealed compositional fluctuations with depth that may be related to depositional events (Supplementary Fig. 5). For instance, at sites A3 and A4 the microbial communities in the oxic zone were more similar to those from the ferruginous zone at around 15–25 and 9–15 cm, respectively, than to the samples of the nitrogenous zone located in between them (Supplementary Fig. 5). This hinted that local depositional events might have entombed parts of the microbial community. We suggest that such events contributed to the enhanced site–site variability in the nitrogenous and ferruginous zones (Supplementary Fig. 4A, C).

### Compositional changes over sediment depth and redox zonation along the Atacama Trench

The observed trends in beta diversity were reflected by distinct phylum-level changes that followed redox zonation and sediment depth (Fig. 2C and Supplementary Fig. 6). Conversely, subsurface peaks of microbial abundance [22] were not reflected in the relative abundance patterns of different phyla (Proteobacteria always split to class level yet referred to as phyla for simplicity), which changed steadily with increasing sediment depth. For instance, some of the dominant groups, Gammaproteobacteria (20.9%; mean read abundance per sediment depth), Bacteroidetes (17.1%), and Thaumarchaeota (12.4%), peaked in relative abundance in the oxic zone and then decreased. Alphaproteobacteria (13.7%) on average had the highest relative abundances in the nitrogenous zone and became rather rare in the ferruginous zone. Planctomycetes rose in relative abundance below the oxic zone from around 9 to 15% and remained at this level below. Atribacteria showed the largest change in relative abundance. After being close to detection limit with an average relative abundance of 0.004% in the oxic and nitrogenous zones, their relative abundance increased approximately ten-fold for every centimeter from the transition to the ferruginous zone, until they became the dominant phylum in deeper sediment sections. Aside from Atribacteria, other lineages such as “*Candidatus* (*Ca*.) Marinimicrobia” (0.1–4.6%), “*Ca*. Woesearchaeota” (1.3–11%) and “*Ca*. Patescibacteria” (0.7–4.7%) increased steadily with increasing sediment depth, while Acidobacteria (~3.4%) and Deltaproteobacteria (~6.1%) showed almost no change. As microbial abundance in the hadal samples of this study fluctuated within less than one order of magnitude with depth [22], these relative abundance patterns resembled absolute abundances estimated by normalizing the data to cell numbers (data not shown).

The directional changes in the microbial community composition with sediment depth and associated redox zonation indicated an active community turnover. Similar succession patterns of Gammaproteobacteria, Thaumarchaeota, Planctomycetes and other major microbial taxa have been found in sediments across the entire oceanic depth range from less than a 100 m water depth to the bottom of the Challenger Deep in the Mariana Trench [9, 24, 25]. Our data indicate that some of these directional changes are associated with redox stratification and thus are an inherent characteristic of cohesive marine sediments.

### Assembly of subsurface phyla in the ferruginous zone in the Atacama Trench

The high spatial resolution of sampling across redox zones at multiple sites provides new insight into the assembly of deeper microbial communities. For example, combining the relative read abundance of Atribacteria (Fig. 2C) with total cell counts [22] (ignoring potential PCR bias and assuming the same 16S rRNA gene copy numbers in this group as in the community on average), we estimate an absolute increase of Atribacteria from a mean of 5.4 × 10^3^ cells cm^−3^ at the upper boundary of the ferruginous zone at 6 cm depth to 1.2 × 10^7^ cell cm^−3^ at 30 cm sediment depth (>2000-fold increase). Excluding mortality, this can be accomplished in 11 generations, and given an estimated sedimentation rate during periods with no mass depositions of approximately 0.05 cm year^−1^ (unpublished data) would have occurred over approximately 500 years. Although the number of generations is a minimum estimate, this timeframe appears to leave relatively little opportunity for diversification, as previously concluded for deeper subsurface sediments [11].

We further note that bioturbation in hadal sediments is mostly limited to meiofaunal infauna and epibenthic amphipods; hence, sediment mixing is unlikely to affect the depth distribution of microbes below the topmost centimeters [38]. As discussed in previous studies, vertical dispersal of microbes by means of active motility is unlikely to play a role in community assembly in cohesive sediments due to energetic constraints and short-distance chemical gradients [9, 39]. As the sediment in both the Kermadec and in the Atacama trench is cohesive [26], and in accordance with previous studies in deeper redox zones [9, 10], we therefore conclude that selection is likely the dominant force controlling community composition and, e.g., giving Atribacteria their dominant role in the ferruginous zone. They appear to grow from a small seed stock that arrives at the sediment surface and survive burial in an inactive state, until oxygen and nitrate are depleted. Other obligate anaerobes in marine sediments may be subject to similar constraints (see also [10]). This implies that there is little diversification potential for obligate anaerobes in hadal trench sediments. Other anaerobic niches in the hadal zone that might have more diversification potential include hydrothermally active sites and the guts of fauna [40, 41]. However, the conditions in guts and hydrothermally active sediments differ from those of cold deep-sea sediments, and these environments therefore harbor very different microbial communities [42, 43]. Hence, the majority of obligate anaerobes must have originated from the overlying water column and come with the necessary adaptations for the increased hydrostatic pressure and other conditions in hadal sediments. We therefore hypothesize that most obligate anaerobes in hadal sediments tolerate but do not prefer hadal pressures.

### Frequency distribution of ASVs and taxonomic affiliation of cosmopolitans along the Atacama Trench

Despite the large number of ASVs present in our dataset, only few were found in all samples from a given redox zone, yet these cosmopolitans tended to account for a large fraction of sequencing reads (Supplementary Fig. 7). This was especially pronounced in the rarefied data from the oxic zone where 365 out of 24,844 ASVs occurred across all oxic samples and comprised over 40% of all reads obtained from this zone, thereby contributing substantially to the similarity between cores and sites within the Atacama Trench (Fig. 2). The majority of these ubiquitous reads originated from ASVs belonging Gammaproteobacteria, Thaumarchaeota, Alphaproteobacteria, and Bacteroidetes (Supplementary Fig. 8). The nitrogenous and ferruginous communities were generally more variable, but ubiquitous ASVs accounted for 15% and 10% of all reads, respectively. In both zones most of the reads originated from ASVs classified as Alphaproteobacteria and Bacteroidetes, with ubiquitous ASVs belonging to *Ca*. Phycisphaerae becoming more abundant in the ferruginous zone.

OTUs with high abundances were previously found to be cosmopolitan in deep-sea sediments [6]. Here, we show that this observation does not change when using ASVs and thus a much finer phylogenetic resolution for the formation of ecological units. The decrease of cosmopolitan ASVs in the nitrogenous and ferruginous zones relative to the oxic zone might be due to dispersal barriers in combination with the small seed-stocks of anaerobes in the upper parts of the sediment, which may lead to a higher level of stochasticity in community assembly in deeper sections. In the oxic zone, physical disturbances lead to resuspension of sediment particles and microbes into the water column [44], where they can be transported along trench axes by bottom currents known to ventilate trenches [45]. Therefore, we suggest that dispersal resulted in greater relative read abundances of ubiquitous ASVs in the oxic zone than in the nitrogenous and ferruginous zones.

### Core microbiomes of each redox zone along the Atacama Trench

To further analyze the overlaps between abundant community members across redox zones, we performed a core community analysis using the toolset of the ampvis2 R package with adjusted cutoff parameters [32] (see Supplementary Material and Methods). This analysis defines ASVs as part of the core microbiome, when they are above 0.05% relative abundance and within the top 50% of all reads. This classified more than 99% of all ASVs as rare biosphere, while the remaining 441 core ASVs accounted for almost half of all reads (Supplementary Fig. 9A). Each redox zone had a distinct core microbiome, with 196, 66, and 91 core ASVs in the oxic, nitrogenous, and ferruginous zones, respectively, comprising 10.6%, 4.6%, and 8.5% of all reads. The three zones had 17 core ASVs in common that comprised 8.2% of all reads. Aside from these common ASVs, the overlaps between the core microbiomes of the oxic and nitrogenous redox zones were greater than those with the ferruginous zone, with the oxic and nitrogenous sharing an additional 60 ASVs (10.3% of all reads). By contrast, the oxic and nitrogenous zones only shared additional 3 (0.4% of all reads) and 8 (1.5% of all reads) ASVs with the ferruginous zone, respectively. Members of the core microbiome are usually abundant species that are present not merely due to immigration or advection but also through growth, and that are of biogeochemical importance [46, 47]. As the phylum-level composition of the core microbiome in each redox zone was mostly congruent with the overall relative abundance of phyla in each zone (Supplementary Fig. 9B), the distinct shifts in the core microbiome compositions between the zones hint at the potential niche spectra of individual phyla associated with each redox zone (see Supplementary Fig. 9). While many of the core ASVs seemed to thrive in both the oxic and in the nitrogenous zone, the conditions of microbial life seemed to change relatively abruptly when entering the ferruginous zone, resulting in the recruitment of deep-biosphere taxa.

### Community composition of Archaea along the Atacama Trench

Around 20% of all ASVs in the universal 16S rRNA gene dataset were classified as Archaea. However, due to known mismatches of universal 16S rRNA gene primer sets with archaeal lineages, in particular the phylum Thaumarchaeota, we thus also sequenced archaea-specific 16S rRNA gene amplicons with the same read depth. This primer set recovered approximately three times more thaumarcheotal ASVs than the universal set (4410 vs 1477) and also showed a better coverage over Euryarchaeota (3728 vs 2032), Crenarchaeota (1884 vs 707, including “*Ca*. Bathyarchaeia”), “*Ca*. Hydrothermarchaeota” (313 vs 101), and Hadesarchaea (71 vs 26). By contrast, the universal 16S rRNA gene dataset contained 17 times more “*Ca*. Woesearchaeota” ASVs (42,582 vs 2512), with this phylum even dominating ASV richness over Thaumarchaeota in the universal dataset. Sequencing the HR horizons with this primer set was only successful for only a small number of the samples. As CR horizons were more successful, we focus on this dataset.

Thaumarchaeota was the overwhelmingly dominant phylum in the archaeal dataset and drove most of the dissimilarity between individual redox zones (Fig. 2B). In the oxic and nitrogenous zones, they contributed up to 99.3% relative abundance, and other lineages, particularly Crenarchaeota, “*Ca*. Hydrothermarchaeota,” Euryarchaeota, and “*Ca*. Asgardaeota,” only increased in relative abundance in the ferruginous zone (Supplementary Fig. 10). Consequently, ordination plots of this dataset only showed a depth gradient to the bottom of the nitrogenous zone, while samples from the ferruginous zone deviated strongly from this gradient (Fig. 2B). Estimates of absolute abundances of the individual archaeal phyla from the universal 16S rRNA gene dataset (Supplementary Fig. 11) showed that the relative depth-wise increase of “*Ca*. Asgardaeota” (0–9.8%) and Crenarchaeota (0–32.9%) in the archaeal dataset reflects their increase in absolute abundance from 1.4 × 10^3^ to 3.4 × 10^5^ “*Ca*. Asgardaeota” per ml sediment and 3.7 × 10^2^ to 4.4 × 10^5^ Crenarchaeota per ml sediment. This suggested possible growth of these lineages in hadal sediments.

Globally, bacterial lineages dominate over archaeal lineages in marine water columns and surface sediments [48, 49]. The only archaeal phylum in these habitats of comparable abundance is Thaumarchaeota. However, in coastal subsurface sediments, archaeal lineages belonging to “*Ca*. Lokiarchaeota” and the Miscellaneous Crenarchaeota Group (here referred to as “*Ca*. Bathyarchaeia”) were found to comprise the majority of intact microbial cells, and Archaea in general contributed significantly to the carbon turnover in these systems [49–52]. These studies showed that recruitment of archaeal strains occurs in the first few centimeters of these sediments but did not provide a more specific location or connection to biogeochemistry. Our data indicated that the enrichment of Crenarchaeota (including “*Ca*. Bathyarchaeia”) and “*Ca*. Asgardaeota” started similarly to that of Atribacteria at the interface of the nitrogenous and ferruginous zones, and was accompanied by an increase in archaeal abundance relative to bacteria. Consequently, both the universal and the archaeal 16S rRNA gene data suggested that the interface between the nitrogenous and ferruginous zones marks the beginning of assembly of subsurface-like microbial communities. This interface also marks the transition from nitrate reduction to iron and/or sulfate reduction as the dominant terminal electron accepting processes. According to existing models, this transition is further associated with a switch in how organic matter is mineralized, with aerobes and denitrifiers being capable of degrading and oxidizing complex organic substrates individually, while a functional division between fermentation and respiration among two sets of organisms is necessary during dissimilatory iron and sulfate reduction [53, 54]. Therefore, we suggest that the distinct differences in microbial communities across the nitrogenous-ferruginous interface are due to the utilization of different electron acceptors and the associated division of labor that causes a rise of fermenters.

### Community composition across hadal, abyssal, and bathyal sediments

To get further insights to factors influencing microbial community composition in hadal sediments, we compared the Atacama Trench to the Kermadec Trench in the less productive western South Pacific, as well as to abyssal and bathyal sites adjacent to these trenches. Kermadec Trench sediments were characterized by deeper oxygen and nitrate penetration depths than in the Atacama Trench (8.5 to >18 cm and 15 to >30 cm, respectively), pushing the ferruginous zone below the sampled sediment horizons at one of the sites (K4 [26], Supplementary Table 1). Consequently, data on the ferruginous zone of the Kermadec Trench were scarce (Supplementary Table 1). In addition, the entire oxic zone was only covered with confidence at site K6, due to potential loss of surface layers at the other stations. Similar to the Atacama Trench, a non-steady state depositional regime in the Kermadec Trench was indicated by fluctuating microbial abundances and visible layering of the sediments.

Sediments from the abyssal plains adjacent to both trenches (A7 and K7) showed even deeper oxygen penetration beyond the measured range of the oxygen profiling lander (>20 cm) and projections indicated that these sediments were oxic across the entire interval analyzed here [26]. In contrast to the trench sites, microbial abundance decayed exponentially with sediment depth and was associated with parallel decreases in TOC content [22]. Conversely, sediment cores from the bathyal (A1) and abyssal (A9) continental slope sites next to the Atacama Trench reached into ferruginous and nitrogenous horizons, respectively, with oxygen penetrating to 1.9 and 6.7 cm, respectively, and nitrate reaching ~6.5 cm at A9. The TOC and microbial abundances showed no clear downcore pattern at these sites [22].

**Fig. 3:**
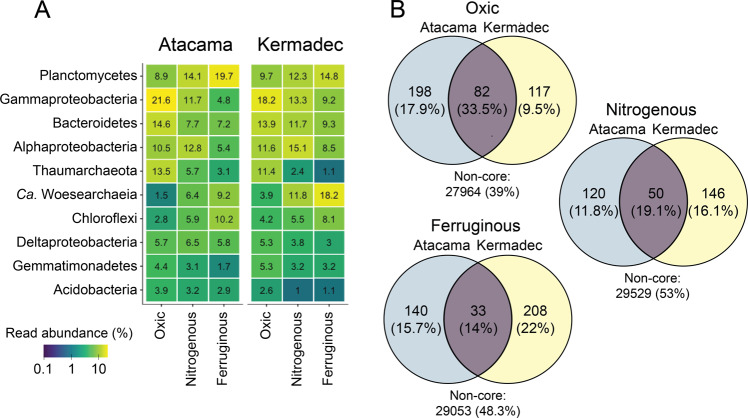
Microbial community composition across trenches. **A** Relative read abundances (%) on phylum/class level of the ten most abundant taxonomic groups (universal 16S rRNA gene data) grouped by individual redox zones in hadal samples of the Kermadec and Atacama trenches. Both the color gradient and the number within the squares indicate the average relative read abundances within the respective sample group. **B** Number of ASVs and relative fractions of total reads constituted by the core microbiomes of the oxic, nitrogenous, and ferruginous zones, respectively, as unique to the Atacama Trench (blue), unique to the Kermadec Trench (yellow), and shared between trenches (purple).

At the phylum level, the hadal communities revealed by universal primers were similar in the two trenches, though the drop in Thaumarchaeota abundance was more sharply located at the oxic-nitrogenous interface in the Kermadec Trench than in the Atacama Trench (Fig. 3A and Supplementary Fig. 12A). The Kermadec Trench also exhibited higher relative abundances of “*Ca*. Woesearchaeota” (18.2% vs 9.2%) in deeper sediment horizons. The archaeal datasets differed more clearly between the two trenches (Supplementary Fig. 12B). While Crenarchaeota reached almost 20% abundance in the Atacama Trench and were detected in the oxic zone, they were essentially absent in the Kermadec Trench. In contrast, “*Ca*. Diapherotrites” (DPANN) contributed up to 28.6% of relative abundance in the deeper sections of the Kermadec sediments but were almost absent in the Atacama Trench. Similar small-scale differences in the relative abundances of microbial phyla were previously observed between the Japan, Izu-Ogasawara and Mariana trenches [24], Mariana and Mussau trenches [55], as well as between the Mariana and Kermadec trenches [25]. Peoples et al. [25] showed that the Kermadec Trench was enriched in Bacteroidetes, “*Ca*. Hydrogenedentes” and Planctomycetes in comparison to the Mariana Trench, while the latter had higher relative abundances of “*Ca*. Marinimicrobia,” Thaumarchaeota, “*Ca*. Woesearchaeota,” and Chloroflexi. Along with this high similarity between trenches on the phylum level, 58% of all OTUs with ≥97% sequence similarity were shared between the Mariana and Kermadec trenches and these shared OTUs comprised over 95% of all 16S rRNA gene amplicon reads [25]. Thus, they concluded that endemism did not cause the community dissimilarity between the trenches and did not occur on the OTU level. Our ASV-based analysis provided a finer phylogenetic resolution [30], had approximately ten-fold higher sequencing depth, and spanned over three redox zones (up to 40 cm sediment depth) instead of the first 10 cm as in the previous study. A core-to-core comparison from A6 and K6 (the only site with an undisturbed sediment surface in the Kermadec Trench), similar to that of Peoples et al. [25], revealed that the sites shared around 8% of all ASVs, yet these shared ASVs accounted for 62% of all obtained reads from the respective samples. A core microbiome analysis for each redox zone (Fig. 3B) further revealed large overlaps in abundant ASVs between the two trenches. Thus, even with our expansion of the analysis we reach a similar conclusion as Peoples and coworkers that endemism must be relatively rare in hadal sediments. However, the overlap between the trenches decreased from the oxic to the nitrogenous and ferruginous zones. While individual redox zones in these two geographically isolated hadal trenches provide similar ecological niches and are to a large extent inhabited by the same abundant ASVs, this decrease of core microbiome overlaps may be driven by enhanced dispersal barriers, as discussed in the previous paragraph.

Unexpectedly, the phylum-level composition and depth distribution at the continental slope (sites A1 and A9) resembled the results from the hadal zone more closely than those from the abyssal plain, with Thaumarchaeota, Alphaproteobacteria, Gammaproteobacteria, and Bacteroidetes decreasing in relative abundance from the oxic to deeper sediment sections, and Chloroflexi increasing with sediment depth. In contrast, at the abyssal sites, the relative abundance of Thaumarchaeota was almost twice as high (A7: 23% K7: 24%) as in the oxic zone of hadal sediments and did not change significantly over sediment depth (Supplementary Fig. 13A). This was particularly pronounced in the archaea-specific dataset, where Thaumarchaeota comprised more than 99% mean read abundance (Supplementary Fig. 13B). However, in contrast to the hadal and continental slope sediments, where increased relative abundance also indicated growth, estimated absolute abundances at the abyssal plain sites generally decreased with sediment depth (Supplementary Fig. 13C). Hence, we propose that the downcore changes in the abyssal plain sediments may reflect differential persistence and survival capabilities rather than growth, similar to conclusions from subsurface sediments [56]. Thus, the conditions for microbial life differ quite fundamentally between abyssal plains and hadal trenches. The similar directional phylum-level changes in the continental slope and hadal sites further supported biogeochemical forcing as a main driver of microbial community composition on the phylum level and suggested that potential effects associated with oceanic depth or hydrostatic pressure are secondary or mainly apply to finer taxonomic levels.

When all sites were compared through PCoA of Bray Curtis dissimilarities, both bathyal and abyssal communities differed from hadal samples (Supplementary Fig. 14A), and ANOSIM confirmed this distinction (*p* > 0.001, *R* = 0.595). The archaea-specific dataset showed similar patterns as the universal dataset except with clearer separation between the two trenches (Supplementary Fig. 14B). This analysis indicated a gradient of microbial community composition with increasing oceanic depth, despite the high similarities of phyla compositions between sediments with similar redox stratifications.

**Fig. 4:**
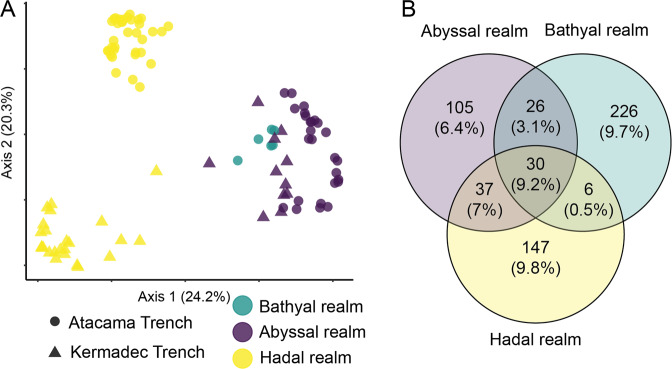
Microbial community composition across benthic realms. **A** Principal coordinate analysis (PCoA) of Bray Curtis dissimilarity across samples from the oxic zone (CR sectioning) from the hadal (yellow), abyssal (turquoise), and bathyal (purple) realms in the Kermadec Trench (triangles) and Atacama Trench (circles) based upon the universal 16S rRNA gene dataset. **B** Number of ASVs and relative fractions of total reads constituted by the core microbiomes of the oxic zones of the hadal (yellow), abyssal (purple), and bathyal (turquoise) realms.

Focusing on oxic horizons across sites, and thus removing the strong effect of the redox gradient, the abyssal samples again showed high similarities to each other and to the bathyal site, while the communities of the hadal zone of each trench clustered separately (Supplementary Fig. 14C, D). Still, ANOSIM indicated a clear separation of hadal from shallower samples (*p* > 0.001, *R* = 0.677) with less variation within the individual groups. These patterns were also reflected in the overlaps of core microbiomes of all oxic samples, in which adjacent realms shared more ASVs (Hadal–Abyssal: 37 ASVs; 7% of all reads; Abyssal–Bathyal: 26 ASVs; 3.1%) than the hadal sites and bathyal site (6 ASVs; 0.5%), in addition to the 30 core ASVs (9.2%) found in all realms (Fig. 4B and Supplementary Fig. 15). The relatively small overlap of core ASVs between the hadal and bathyal sites indicates a gradient in community composition of the oxic zone across depth realms with respect to the more abundant members. Factors that could impose such a barrier on core microbiome constituents and benthic microbial communities in general are discussed in the next section.

### Factors controlling community composition in hadal vs bathyal and abyssal sediments

Previous studies on hadal trench sediments suggested that geochemical factors had a stronger impact on community composition than, for instance, hydrostatic pressure [24]. In this section we aim to test previous hypotheses by determining how well TOC concentration and redox zonation explain variation in the microbial communities. We start by excluding potential confounding effects of oceanic depth and geographic isolation by focusing on the Atacama Trench.

#### Dissimilarity within a trench

In the Atacama Trench, the ordinations of Bray Curtis dissimilarity already hinted that factors associated with sediment depth and redox zonation were the driving forces of microbial community composition (Fig. 2A, B). Previously, it was shown that variation in TOC concentration (ranging from 0.3 to 1.5%) was one of the best predictors of community variation in abyssal and bathyal sediments [6, 57]. In the Atacama Trench, TOC concentration decreased from the northernmost site A10 (1.44 ± 0.35%; downcore average ± SD) to the southernmost A6 (0.44 ± 0.09%; A6) and this decrease coincided with a decrease in metabolic activity along the trench axis [22, 26]. TOC concentrations also fluctuated with increasing sediment depth at each site, most pronouncedly at sites A2 and A10, where values peaked at around 9 cm depth and varied downcore between 0.3–0.9 and 0.7–2.0%, respectively [22].

**Fig. 5:**
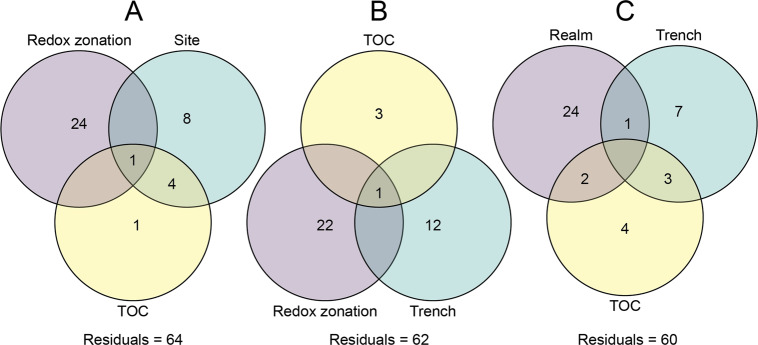
Factors controlling microbial community composition. Variation partitioning (%) of Hellinger-transformed ASV counts between explanatory factors from hadal samples of the Atacama Trench (**A**), across hadal samples from the Atacama and Kermadec trenches (**B**), and between oxic samples of all oceanic realms associated in the two trench systems (**C**).

We delineated the effects of redox zonation from site–site variation and TOC concentration on microbial community composition using variation partitioning on Hellinger-transformed ASV counts (Fig. 5A and Supplementary Fig. 16A). The unique fraction of variation statistically explained by TOC was very low (1%, *p* = 0.014) yet had a large overlap of 4% with the site-to-site variability. This overlap disappeared completely when A10 was excluded, hinting that much of this trend was driven by high TOC concentrations at this single site. Thus, TOC was a poor predictor of microbial community composition in the Atacama Trench, despite the high downcore fluctuations and the broader concentration range along the trench axis than across all locations of the global dataset on abyssal and bathyal surface sediment of Bienhold et al. [6]. Instead, redox zonation explained the largest unique fraction of variation (24%, *p* < 0.001) of all analyzed variables.

The effect of redox chemistry on overall community composition was previously assessed for sulfidic and methanic zones [11, 13] and found to be secondary to sediment depth and age. Data on the transition between the oxic and nitrogenous zone have thus far been limited to clone libraries [14]. Our data indicate that redox stratification has a strong impact on community composition based on (a) the overall relative abundance changes of phyla in particular between the nitrogenous and ferruginous zone (see Fig. 1C), (b) the abundance peaks of functional key groups in the core microbiome (see Supplementary Fig. 9), and (c) the variation of community composition explained by sediment depth (2%) and redox zonation (9%), with a notable covarying overlap of 15% (Supplementary Fig. 16A). In contrast to the aforementioned sulfidic/methanic sediments and our abyssal plain sediments, sediment depth in hadal sediments does not necessarily reflect sediment age, and community changes are associated with growth rather than environmental filtering. Hence, it remains an open question why and over what timeframe the community composition changes within individual redox zones in deep-sea sediments.

#### Dissimilarity between trenches

Similar to changes in community composition within a trench, the distinction between trenches was hypothesized to be driven by variable amounts of TOC [24, 25]. The Atacama and Kermadec trenches were good model systems to test this hypothesis due to the contrasting primary productions in the overlying water column and the related differences of benthic TOC concentrations between each trench (Atacama: 0.64 ± 0.42% Kermadec: 0.27 ± 0.09). With TOC, redox zonation, and trench as explanatory variables, the strongest factor in explaining the variation of community composition was again redox zonation (22%; *p* < 0.001), without any collinear overlap to either the trench–trench factor or TOC concentrations (Fig. 5B and Supplementary Fig. 17). This further emphasized that redox zonation and downcore gradients are ubiquitous forces in structuring hadal microbial communities. A small part of the variation between Kermadec and Atacama trenches was associated with TOC content (*p* = 0.018) but with little impact (3%), while the trench variable explained 12% (*p* < 0.001) in variation partitioning. This variable covers a variety of potential controlling factors like geographic distance [6], oceanic currents [5], mineralogy [58], oceanic depth [25], and sediment age [11]. Consequently, the exact reasons why different trenches have different communities remain unresolved.

#### Dissimilarity between realms

Finally, we tested to what extent the different oceanic realms (hadal–abyssal–bathyal), TOC concentrations, and trench–trench variability explained the variation of microbial abundances in our data (Fig. 5C and Supplementary Fig. 16C). To exclude the effect of the redox gradient, we selected oxic samples for this analysis. The largest amount of unique variation was explained by the categorical distinction between oceanic realms (24%, *p* < 0.001), while the unique fraction of variation explained by TOC (4%, *p* < 0.001) again was small. The factors associated with oceanic realms explained more variation in community composition than the vast geographic distance between the two trenches (7%, *p* < 0.001) and all associated biogeochemical factors. This confirmed the observed trends in the core microbiome analyses and ordination plots (Fig. 4A, B).

While our results indicate that realm (i.e., hadal vs abyssal) is an important factor influencing benthic microbial community composition, the underlying mechanisms of this effect are not clear. What is more clear is that oceanic depth had a stronger impact on microbial community composition than either potential limitations on microbial dispersal between the two trenches or differences in the primary productivity of their respective overlying water columns. Community variability could reflect adaptions of certain microbial strains to the hydrostatic gradient [59, 60]. However, aside from this physical gradient, several other factors with potentially stronger selective effects differ between the oceanic realms. Thus, the supply of organic carbon from the overlying water column generally decreases with increasing depth and is accompanied by an increase of recalcitrance [16, 61]. In addition, the lateral transport of sediment via mass wasting events has the potential of burying fresh organic material into deeper sediment sections and thereby affecting community composition [17, 62]. Other potential selective forces in the hadal zone include enrichments of heavy metals and persistent organic pollutants [63]. The effect of fauna on the microbial community composition may also be more similar between adjacent realms yet has so far not been explored. We therefore propose that a combination of multiple gradual and categorical differences led to the higher overlaps of core communities between adjacent oceanic realms, rather than the increasing hydrostatic pressure alone.

## Conclusion

Our analyses revealed that benthic microbial communities were strikingly similar in the horizontal direction on the 430-km-long transect along the Atacama Trench axis, despite the characteristic irregular depositional regime in the hadal realm. While we found some indications of entombments of communities that might have caused some site-to-site variability, community composition was principally linked to redox stratification. In particular, the interface between the nitrogenous and ferruginous zones marked an distinct boundary where taxa unique to the deep biosphere were recruited. The observed taxonomic shift probably resulted from a metabolic division during organic carbon mineralization between fermentation and respiration. The gradients in community composition within redox zones in hadal sediments were likely not related to sediment age, hence factors driving these gradients require further attention in future studies.

We found several cosmopolitan ASVs in each redox zone that were present in high abundances along the entire Atacama Trench axis and widely shared between both trenches. This was particularly the case for aerobic communities, where microbes have a longer residence time and thus a larger spreading potential than in deeper sections. Contrary to previous hypotheses, our data showed that from a statistic perspective, organic carbon enrichment cannot explain why hadal communities are dissimilar to communities in adjacent abyssal and bathyal settings. Other selective forces that are likely associated with ocean depth and/or trench topography must cause this dissimilarity and explain why adjacent realms share more high-abundance core microbiome ASVs.

Microbes must enter the hadal sediment through deposition, and reach their preferred niche through burial, which leaves very limited room for evolutionary adaptions. Hence, we deduce that the majority of anaerobic microbes in hadal sediments are tolerant to hadal hydrostatic pressure rather than preferring it. We further hypothesize that hadal communities differ from adjacent shallower settings due to changes in multiple environmental factors such as the presence and activity of fauna and compositional differences in the organic carbon pool.

## Supplementary information

Supplementary information
