# Supplementary material for: Microbial community structure in hadal sediments: high similarity along trench axes and strong changes along redox gradients
Source: ISME J. 2021 Jun 8;15(12):3455–67. doi: 10.1038/s41396-021-01021-w (PMC8629969; doi:10.1038/s41396-021-01021-w)

Supplements

Supplementary Material and Methods

Sampling sites and core collection

The Kermadec and Atacama trenches were sampled along their elongated axes. Sediment was collected from four hadal sites in the Kermadec Trench (Maximum depth 10 047 m; R/V Tangaroa cruise TAN1711 2017) and six hadal sites in the Atacama Trench (Maximum depth 8055 m: R/V Sonne cruise SO261 2018) (Supplement table 2). To explore differences between hadal and abyssal microbial communities, an abyssal site was visited on the subducting plate side of each trench, and in the Atacama Trench one abyssal and one bathyal site on the continental shelf were also sampled. In the Atacama Trench, all sediment cores were obtained using a multicorer and had clear overlying water in the core liners, indicating that the sediment surfaces were only minimally disturbed by coring. In the Kermadec Trench, undisturbed cores were obtained from sites 4 and 6 using an autonomous benthic lander and a multicorer, respectively, while other samples from site 3, 4, 5 and 7 were obtained by sub-coring a 50x50 cm boxcorer. Porewater chemistry indicated losses of the upper ≤1, ≤1, and 5-7 cm at site 3, 4, and 5, respectively, whereas at site 7 the samples showed no sign of disturbance.

Sediment core processing

The sediment cores were sectioned in a 3 °C cold room immediately upon shipboard arrival, using sterilized utensils and two different sectioning schemes with coarser and finer spatial resolution. The coarser resolution scheme (referred to as CR) followed the guidelines of the ABYSS project with

triplicate cores sectioned in 0–1 cm, 1-3 cm, 3-5 cm, 5-10 cm, 10-15 cm, 15-30 cm and 30 – bottom of the core intervals, placed into sterile plastic bags (VWR), homogenized and immediately frozen to –80 °C. The higher resolution (HR) scheme was included to resolve changes in community composition along the relatively steep redox gradients of the Atacama Trench and involved one core per site sectioned in 1 cm horizons down to 10 cm, 2.5 cm down to 30 cm, followed by 5 cm slices until the bottom of the cores. Subsamples from each horizon were taken using sterile, cut off syringes and stored in PCR graded Eppendorf Tubes at –80 °C.

Biogeochemical categorization

In parallel to the molecular work of this study, a multitude of studies from different fields was conducted on either the same sites or even same samples. Analyses included prokaryotic and viral counts [1], oxygen profiles [2], and porewater chemistry, the results of which serve as metadata for this study. The oxygen profiles and porewater analyses were used to assign the sediment horizons of this study to the redox categories oxic, nitrogenous, or ferruginous as defined by Canfield and Thamdrup (2009) and exemplified in Supplement Figure 1. Horizons were classified as oxic if their mid-depth was shallower than the oxygen penetration depth [2]. Similarly, deeper horizons were classified as nitrogenous if nitrate was present until the mid-depth or deeper (Supplement Table 1), while deeper horizons, characterized by accumulation of Fe^{2+} in the porewater were classified as ferruginous (Supplement Figure 1). A sulfidic zone was not reached at any of the stations. The porewater chemistry data can be accessed in the Pangaea repository (www.pangaea.de) under project title HADES-ERC.

DNA extraction:

In samples from the CR scheme, DNA was extracted from 10 g of sediment using the DNeasy PowerSoil Max Kit. The only modification to the manufacturer's protocol was 20 minutes agitation during the first cell lysis step instead of 10 min of vortexing. Samples from the HR scheme were extracted using a downscaled approach (DNeasy PowerSoil Kit) with approximately 0.25 g of Sediment per extraction and 15 min of gentle vortexing instead of agitation.

Amplicon generation and sequencing

PCR amplification:

Amplicon sequencing of general and archaea specific 16S rRNA gene was conducted at Genoscope, C.E.A, Institut de Biologie François Jacob, as described in Brandt *et al.*, 2019. Briefly, universal 16S rRNA amplicons were generated using the 515F-Y (5′- GTGYCAGCMGCCGCGGTAA-3′) and 926R (5′- CCGYCAATTYMTTTRAGTTT-3′) primers [4]. The PCR reaction was conducted in triplicates on each sample in order to account for intra-sample variations of the first few PCR cycles (98 °C for 30 s; 25 cycles of 10 s at 98 °C, 30 s at 53 °C, 30 s at 72 °C; and 72 °C for 10 min). Each PCR mix contained 2.5 ng or less of DNA template, 0.4 µM concentration of each primer, 3% DMSO and 1× Phusion Master Mix (company).

A similar procedure was used for amplifying the V4–V5 hypervariable region of the archaeal 16S rRNA gene, using an equimolar pooled mixture of primer variants for position 517F (5'-GCCTAAAGCATCCGTAGC; GCCTAAARCGTYCGTAGC; GTCTAAAGGGTCYGTAGC; GCTTAAAGNGTYCGTAGC; GTCTAAARCGYYCGTAGC-3') and a single reverse primer 958R (5'-CCGGCGTTGANTCCAATT-3'[5]. Again, triplicate PCR reactions were performed, using 2.5 ng or less of total DNA template with 0.5 µM final concentration of each primer, 3% of DMSO, 0.175 mM final concentration of dNTPs, and 1X Advantage 2 Polymerase Mix (Takara Bio, Kusatsu,

Japan) with slightly different cycling conditions than for the general 16S rRNA (95 °C for 3 m; 25 cycles of 30 s at 95°C, 45 s at 57 °C, 60 s at 68 °C; and 68 °C for 2 min).

Clean-up :

The PCR products were pooled and cleaned using 1X AMPure XP beads, and amplicon lengths were checked with the DNA High Sensitivity LabChip kit (Agilent Technologies, Santa Clara, CA, USA). Subsequently the concentration of the purified PCR products was quantified with a Qubit fluorometer (Invitrogen, Carlsbad, CA, USA).

Amplicon library preparation

From each purified PCR product pool, one hundred ng were end-repaired, A-tailed and ligated to Illumina adapters on a Biomek FX Laboratory Automation Workstation (Beckman Coulter, Brea, CA, USA). Afterwards, each library was amplified using a Kapa Hifi HotStart NGS library Amplification kit (Kapa Biosystems, Wilmington, MA, USA) and purified again with 1X AMPure XP beads.

Sequencing library quality control

Libraries were quantified with both a Quant-iT dsDNA HS assay kits using a Fluoroskan Ascent microplate fluorometer (Thermo Fisher Scientific, Waltham, MA, USA) and qPCR with the KAPA Library Quantification Kit for Illumina Libraries (Kapa Biosystems, Wilmington, MA, USA) on a MxPro instrument (Agilent Technologies, Santa Clara, CA, USA). A high-throughput microfluidic capillary electrophoresis system (LabChip GX, Perkin Elmer, Waltham, MA, USA) was used to assess the library profiles.

Sequencing procedures

The concentrations of all libraries were normalized to 10 nM by addition of 10 mM Tris-Cl (pH 8.5) and clusters generated according to the Illumina Cbot User Guide (Part # 15006165). Paired-end sequencing (2×250bp) of the libraries was performed on either the HiSeq 4000 or HiSeq 2500

instruments (Illumina, San Diego, CA, USA). In order to enhance the sequence quality and counteracting the low contrast of the first few bp due to adapters and primers, the loading concentration of the libraries was reduced from 12-14 pM to 8-9 pM, while PhiX DNA spike-in was increased (20% instead of 1%). Sequencing was otherwise performed according to the HiSeq 4000 System User Guideline (Part # 15011190) and the HiSeq 2500 System User Guideline (Part # 15035786).

Demultiplexing, ASV generation, taxonomic classification, phylogeny reconstruction and filtration of negative controls

Due to the aforementioned library preparation, each of the two paired-end read files contained a mixture of forward and reverse reads. We sorted, renamed, and primer clipped these via the combination of an in-house script and the cutadapt tool. Subsequently the identification and removal of reads without an associated forward or reverse counterpart was conducted using BBmap repair.

ASV tables were generated separately for each sequencing-run and merged with mergeSequenceTables (repeats = "sum") before chimera identification (removeBimeraDenovo, method = "consensus"), all using the DADA2 package (v1.10.1) [6]. Taxonomy was assigned to the ASVs via the Naïve Bayesian-based RDP classifier as implemented within the DADA2 package [7]. ASVs were exported to a FASTA file (seqinr R package version 3.4-5) and a de novo alignment calculated with MAFFT using default parameters [8, 9]. Subsequently, a phylogenetic tree was constructed with FastTree, using the default model in double precision mode due to the low sequence dissimilarity [10].

Negative controls were taken and treated equally to the samples along the entire laboratory process. This includes sampling procedure (containers for the sediment samples), DNA extractions and PCRs. Ultimately, we pooled all negative control samples together and removed the thereby identified

contaminating ASVs from the entire ASV table via prevalence (threshold = 0.5) with the decontam R package version 1.2 [11].

Microbiome analyses

Biostatistical analyses and visualizations of the microbiome data were conducted with the phyloseq and ampvis2 packages in RStudio [12, 13]. Prior to calculating ordinations, ANOSIM, variation partitioning, and RDA, cumulative sum scaling normalization was applied using the metagenomeSeq R package [14]. For relative abundance, absolute abundance and core-microbiome analyses we rarefied our data to even sampling depth.

Ordination plots were produced using principle components analysis of Bray Curtis dissimilarities and distinctions between groups tested using the ANOSIM implementation in the vegan package [15]. The coherence of Bray Curtis dissimilarity with phylogenetic dissimilarity metrics (weighted and unweighted UniFrac) was assessed by comparing the resulting PCoA plots. We used the microbiome extension (release 3_9) for phyloseq to calculate the deviations (divergence function) from the mean Bray Curtis similarities (1 – dissimilarity) within each CR and HR (Supplement Figure 3 A B) horizon along the Atacama Trench axis and the variation within one site (Supplement Figure 3 C).

Core-microbiome analyses were conducted in the ampvis2 environment, with a relative frequency cutoff (cut_f = 50) and relative abundance cutoff (cut_a = 0.05) to account for the large variability in sediments and fine phylogenetic resolution provided by ASVs [16].

We z-scored our metadata and Hellinger transformed the individual ASV counts for subsequent variation partitioning and redundancy analysis with the vegan package [17].

References

1. Schauberger C, Middelboe M, Larsen M, Peoples LM, Bartlett DH, Kirpekar F, et al. Spatial

variability of prokaryotic and viral abundances in the Kermadec and Atacama Trench regions. *Limnol Oceanogr* 2021; e-pub ahead of print 28 February 2021; https://doi.org/10.1002/lno.11711

2. Glud RN, Berg P, Thamdrup B, Larsen M, Stewart HA, Jamieson AJ, et al. Hadal trenches are dynamic hotspots for early diagenesis in the deep sea. *Commun Earth Environ* 2021; **2**: 1–8.

3. Brandt MI, Trouche B, Henry N, Liautard-Haag C, Maignien L, de Vargas C, et al. An Assessment of Environmental Metabarcoding Protocols Aiming at Favoring Contemporary Biodiversity in Inventories of Deep-Sea Communities. *Front Mar Sci* 2020; **7**: 836080.

4. Parada AE, Needham DM, Fuhrman JA. Every base matters: Assessing small subunit rRNA primers for marine microbiomes with mock communities, time series and global field samples. *Environ Microbiol* 2016; **18**: 1403–1414.

5. Huse SM, Mark Welch DB, Voorhis A, Shipunova A, Morrison HG, Eren AM, et al. VAMPS: A website for visualization and analysis of microbial population structures. *BMC Bioinformatics* 2014; **15**: 41.

6. Callahan BJ, McMurdie PJ, Rosen MJ, Han AW, Johnson AJA, Holmes SP. DADA2: High-resolution sample inference from Illumina amplicon data. *Nat Methods* 2016; **13**: 581–583.

7. Wang Q, Garrity GM, Tiedje JM, Cole JR. Naïve Bayesian classifier for rapid assignment of rRNA sequences into the new bacterial taxonomy. *Appl Environ Microbiol* 2007; **73**: 5261–5267.

8. Charif D, Lobry JR. SeqinR 1.0-2: A Contributed Package to the R Project for Statistical Computing Devoted to Biological Sequences Retrieval and Analysis. 2007. Springer, Berlin, Heidelberg, pp 207–232.

9. Katoh K, Standley DM. MAFFT multiple sequence alignment software version 7:

Improvements in performance and usability. *Mol Biol Evol* 2013; **30**: 772–780.

10. Price MN, Dehal PS, Arkin AP. Fasttree: Computing large minimum evolution trees with profiles instead of a distance matrix. *Mol Biol Evol* 2009; **26**: 1641–1650.

11. Davis NM, Proctor DiM, Holmes SP, Relman DA, Callahan BJ. Simple statistical identification and removal of contaminant sequences in marker-gene and metagenomics data. *Microbiome* 2018; **6**: 226.

12. McMurdie PJ, Holmes S. Phyloseq: An R Package for Reproducible Interactive Analysis and Graphics of Microbiome Census Data. *PLoS One* 2013; **8**.

13. Andersen K, Kirkegaard R, Karst S, Albertsen M. ampvis2: an R package to analyse and visualise 16S rRNA amplicon data. *bioRxiv* 2018; 299537.

14. Paulson J. metagenomeSeq: Statistical analysis for sparse high-throughput sequencing. *BioconductorJp* 2014; 1–20.

15. Oksanen J, Blanchet FG, Friendly M, Kindt R, Legendre P, Mcglinn D, et al. (2020) vegan: Community Ecology Package, R package version 2.5.7.

16. Saunders AM, Albertsen M, Vollertsen J, Nielsen PH. The activated sludge ecosystem contains a core community of abundant organisms. *ISME J* 2016; **10**: 11–20.

17. Buttigieg PL, Ramette A. A guide to statistical analysis in microbial ecology: A community-focused, living review of multivariate data analyses. *FEMS Microbiol Ecol* 2014; **90**: 543–550.

Supplementary Tables

Supplement Table 1:

Sampling positions, maximum water depths, oxygen penetration depths (OPD) from Glud *et al*., (2021) and nitrate penetration depths (NPD) of each site in the Kermadec Trench and Atacama Trench. The * indicate samples were taken from a Boxcorer. The ** shows an estimated oxygen penetration based on an empirical relation between DOU and OPD while *** imply that the value is based on only one observation, the remaining profiles did not reach anoxia (see Glud *et al*., 2021)

TAN1711 Kermadec Trench

Site	Latitude	Longitue	water depth (m)	OPD (cm)	NPD (cm)
K3	30° 22.84' S	176° 38.48' W	9540	8.6 ± 0.5	17.5*
K4	31° 08.41' S	176° 48.48' W	9300	20.7**	> 30*
K5	31° 56.14' S	177° 17.48' W	10010	8.9 ± 0.1	4-6*
K6	32° 08.93' S	177° 23.91' W	9555	11.5 ± 0.3	15
K7	32° 11.22' S	176° 33.66' W	6080	26.4**	> 30

SO261 Atacama Trench

Site	Latitude	Longitue	water depth (m)	OPD (cm)	NPD (cm)
A1	23° 48.72' S	70° 50.04' W	2560	1.9***	6
A2	21° 46.86' S	71° 12.48' W	7995	3.2 ± 0.1	8
A3	23° 02.94' S	71° 18.12'W	7915	2.6 ± 0.1	6
A4	23° 21.78' S	71° 20.60' W	8085	3.4 ± 0.4	8
A5	23° 49.02' S	71° 22.32' W	7770	4.0 ± 0.2	8
A6	24° 15.96' S	71° 25.38' W	7720	4.1 ± 0.3	8
A7	22° 56.22' S	71° 37.08' W	5500	21.7***	> 25
A9	20° 19.97' S	70° 58.70' W	4050	6.2 ± 0.5	> 15
A10	20° 19.14' S	71° 17.46' W	7770	3.1 ± 0.3	6

Supplement Table 2:

Summary of successfully sequenced universal and archaea-specific 16S rRNA genes in samples of from each trench and redox zone.

**All Samples:**						
Zonation	Atacama HR universal 16S	Atacama CR universal 16S	Kermadec CR universal 16S	Atacama HR archaea 16S	Atacama CR archaea 16S	Kermadec CR archaea 16S
Oxic	47	118	41	34	63	47
Nitrogenous	37	71	7	17	38	14
Ferruginous	81	41	11	32	36	2
**Hadal Samples:**						
Zonation	Atacama HR universal 16S	Atacama CR universal 16S	Kermadec CR universal 16S	Atacama HR archaea 16S	Atacama CR archaea 16S	Kermadec CR archaea 16S
Oxic	20	70	27	15	36	32
Nitrogenous	25	53	7	10	25	14
Ferruginous	71	35	11	23	32	2

Supplementary Figures

Supplement Figure 1:

Example profiles from Atacama site 2 of (A) in situ oxygen data of Glud *et al*., (2021) and concentrations of reduced iron and nitrate (unpublished data) (B) microbial abundance and total organic carbon content (TOC %) from Schauberger *et al*., (2021). The separation between oxic, nitrogenous and ferruginous zones is indicated by the grey shading.

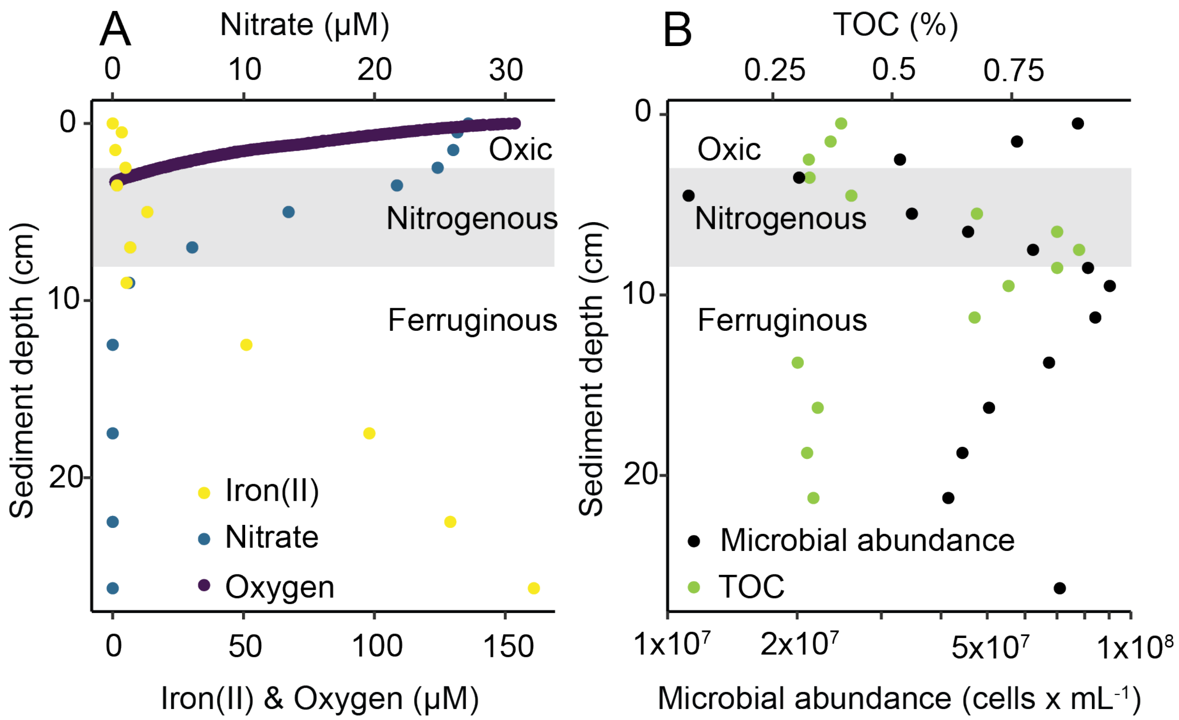

Supplement Figure 2:

Descending number of obtained reads from each individual ASV of the universal 16S rRNA gene **(A)** and archaea specific 16S rRNA gene **(C)** data. Read depths of each sample of the universal 16S rRNA gene **(B)** and archaea-specific 16S rRNA gene **(D)** data.

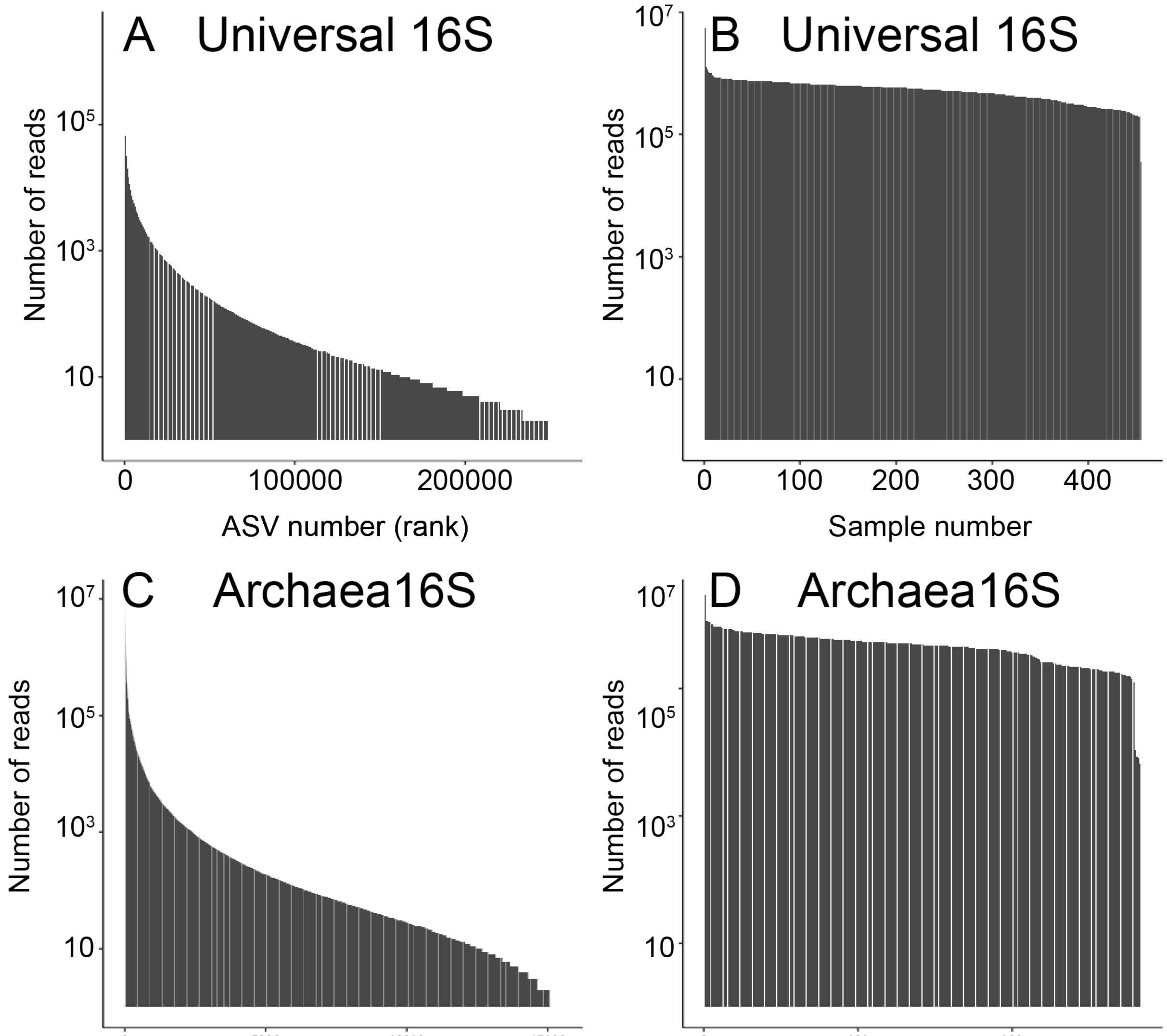

Supplement Figure 3:

(A) ANOSIM statistic over sediment depth. Values larger than 0 indicate that dissimilarity between two different sediments horizons was larger than within one sediment horizon. Values below 0 indicate the opposite. **(B)** Bray Curtis similarity (1 - dissimilarity) matrix over sediment depth and redox zonation

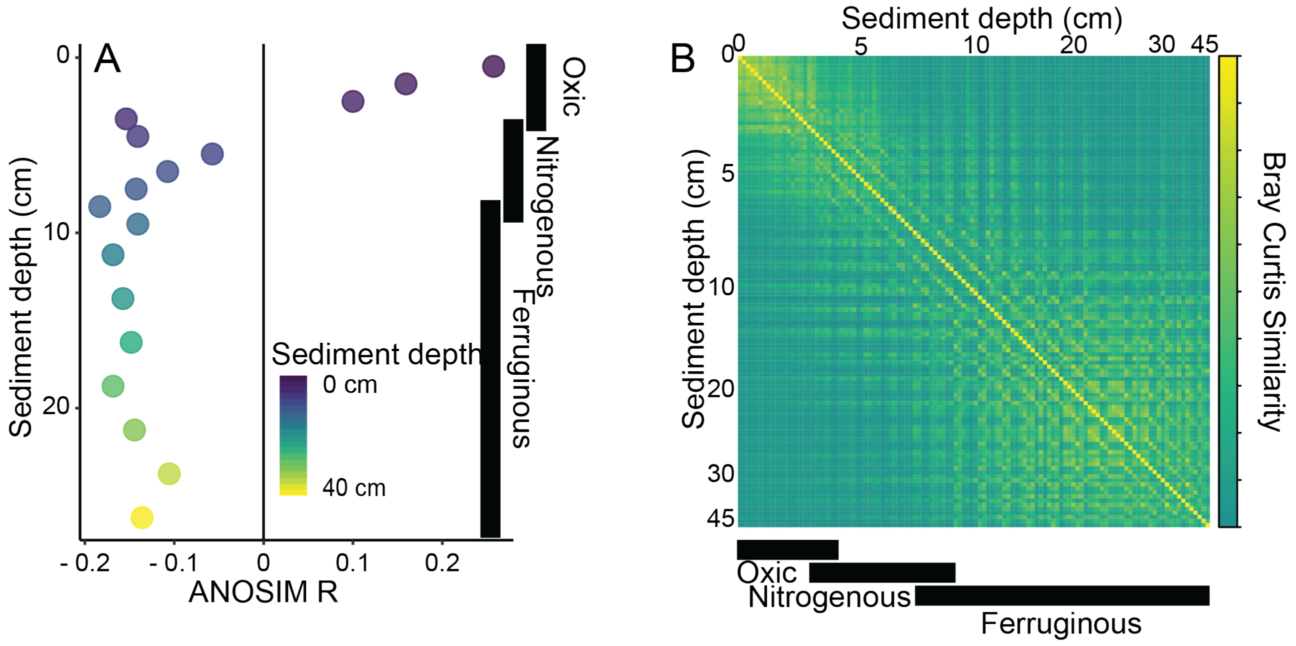

Supplement Figure 4:

(A) Average Bray Curtis similarity (1 - dissimilarity) of all hadal samples from the same CR sediment horizon along the entire Atacama Trench axis. **(B)** Change of similarity between individual hadal sediment sections (HR horizons) along the entire Atacama Trench axis. Different colors represent the different sites. **(C)** Similarity within the three CR samples of the same sediment horizon of each hadal site. Different colors represent the different sediment cores.

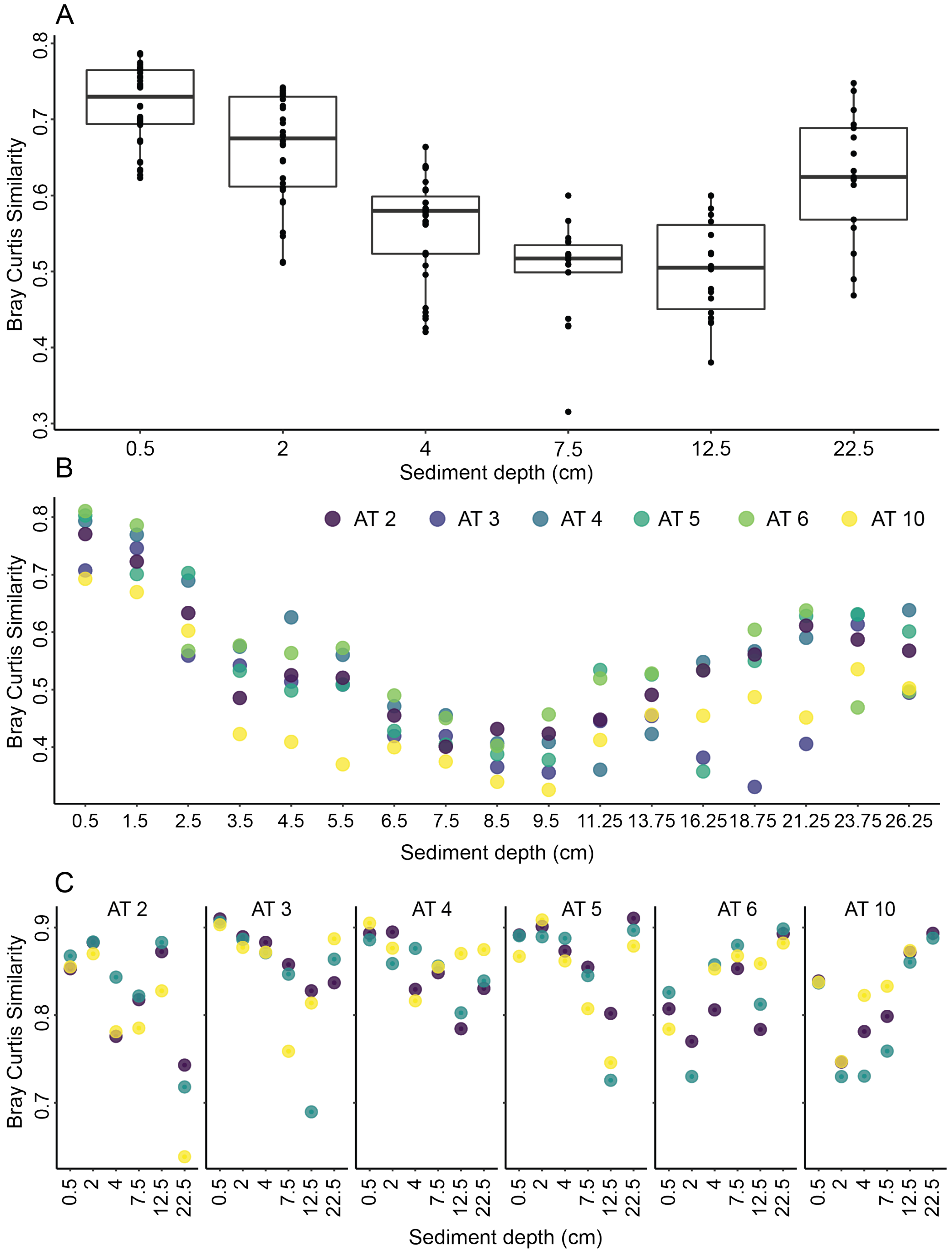

Supplement Figure 5:

Bray Curtis similarity (1 - dissimilarity) matrices of each individual HR sectioned core in the Atacama Trench, with increasing sediment depth on both axes.

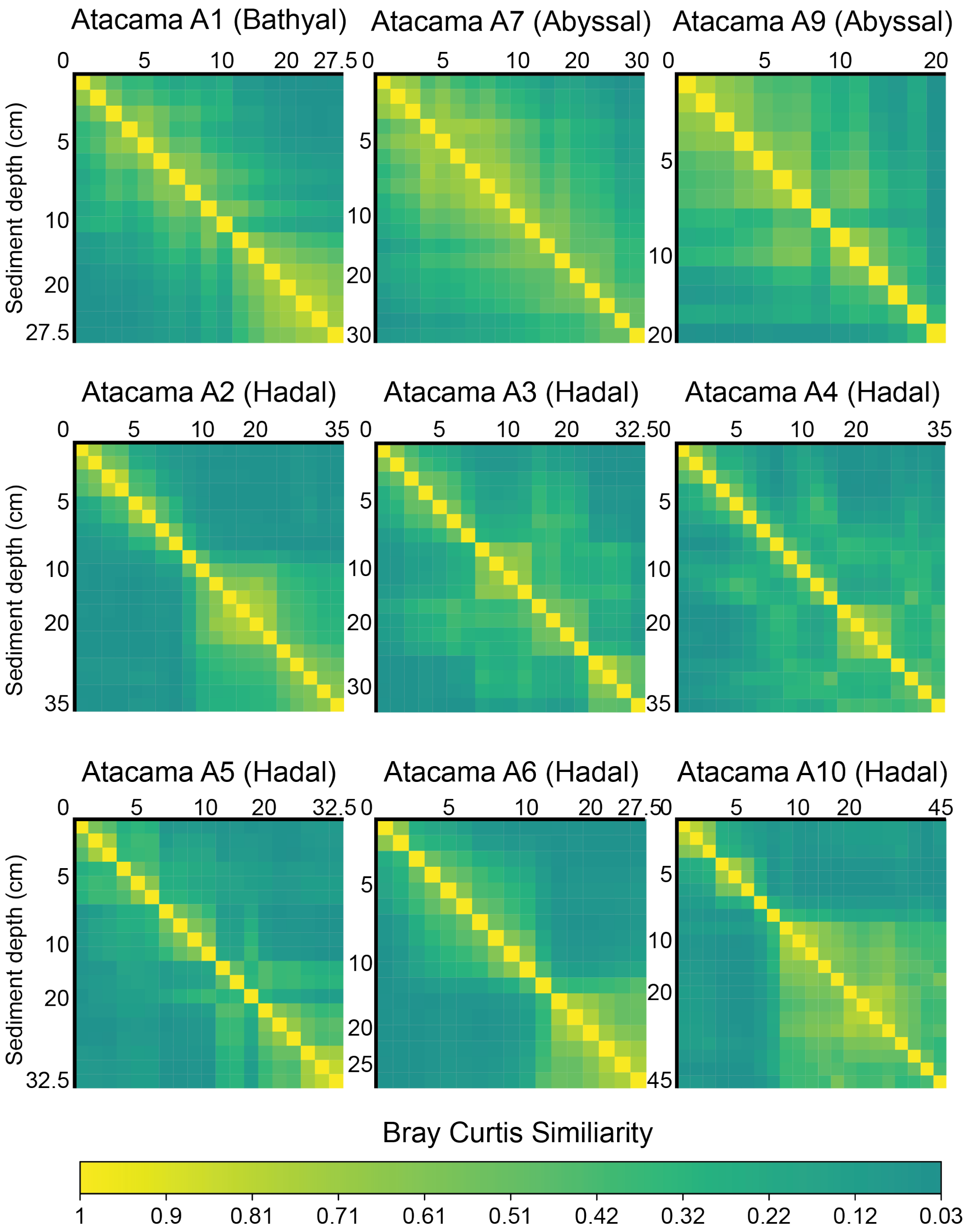

Supplement Figure 6:

Depth profiles of the relative read abundances (%) of the 15 most abundant phyla/classes based on the universal 16S rRNA gene data. Color indicates the hadal site corresponding to each data point.

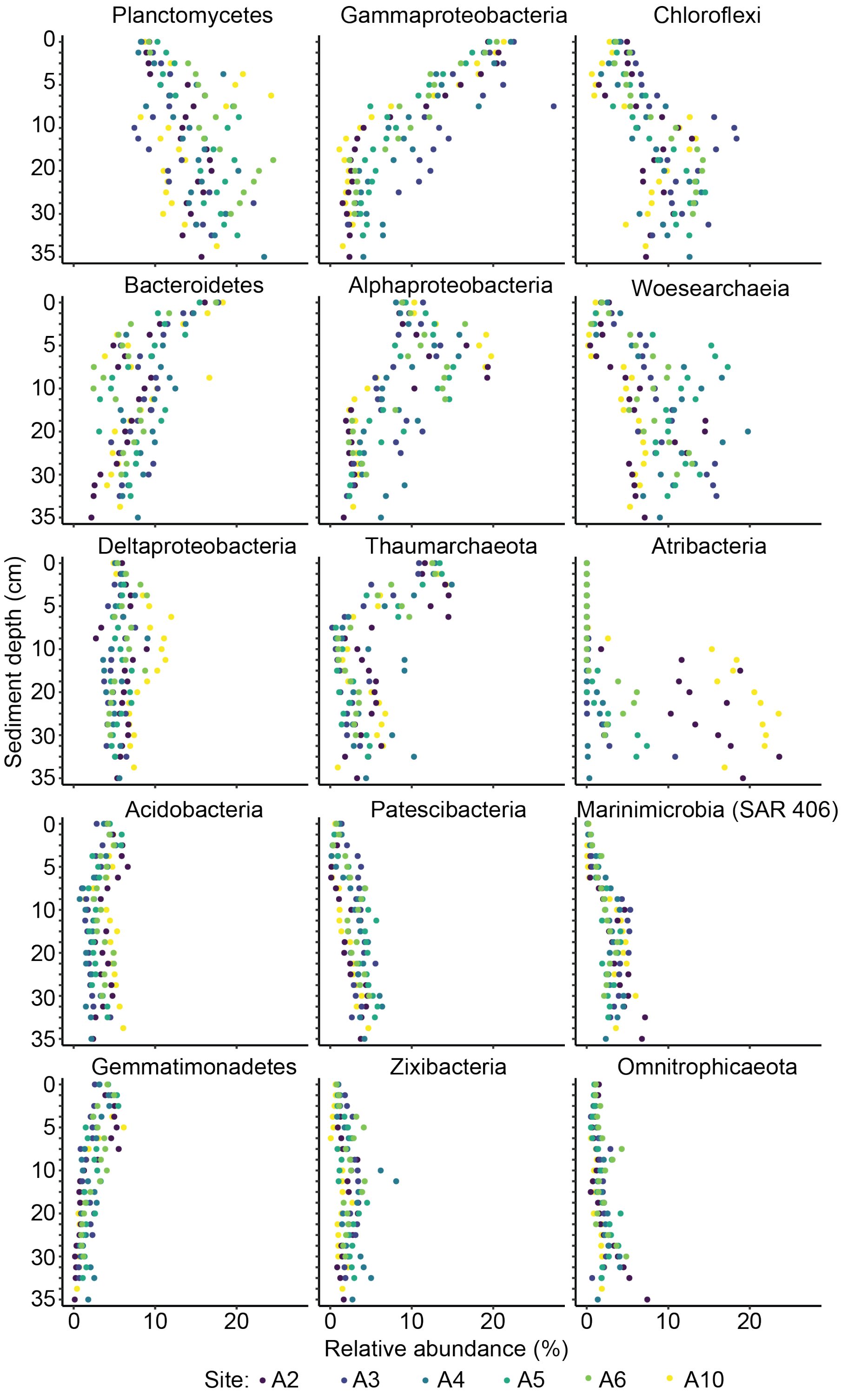

Supplement Figure 7:

Frequency distribution (turquoise) of ASVs across all hadal samples from the Atacama Trench and the relative read abundances (purple).

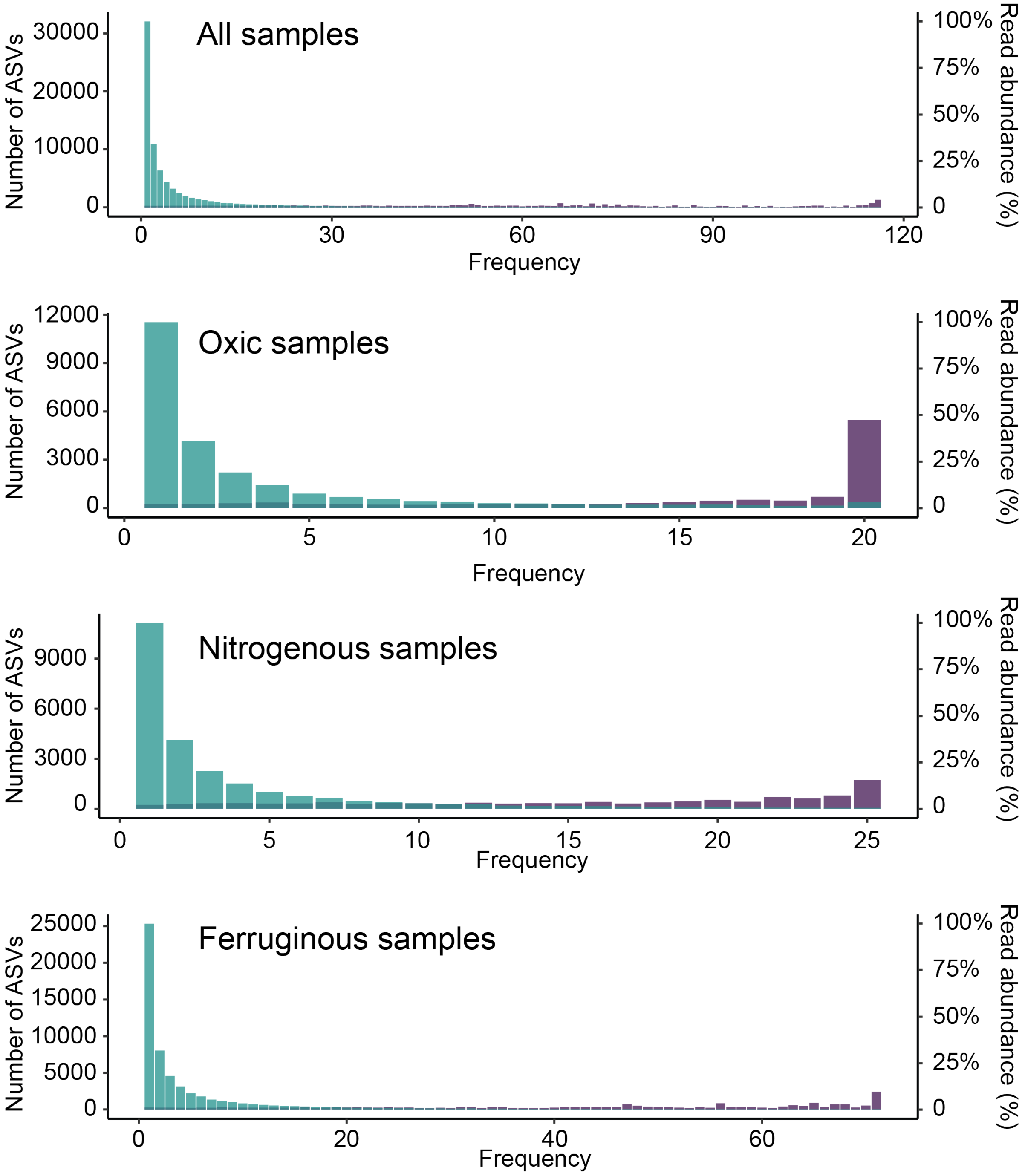

Supplement Figure 8:

Relative abundances of phyla/classes of ubiquitous ASVs (those that occur in all samples) from each redox zone.

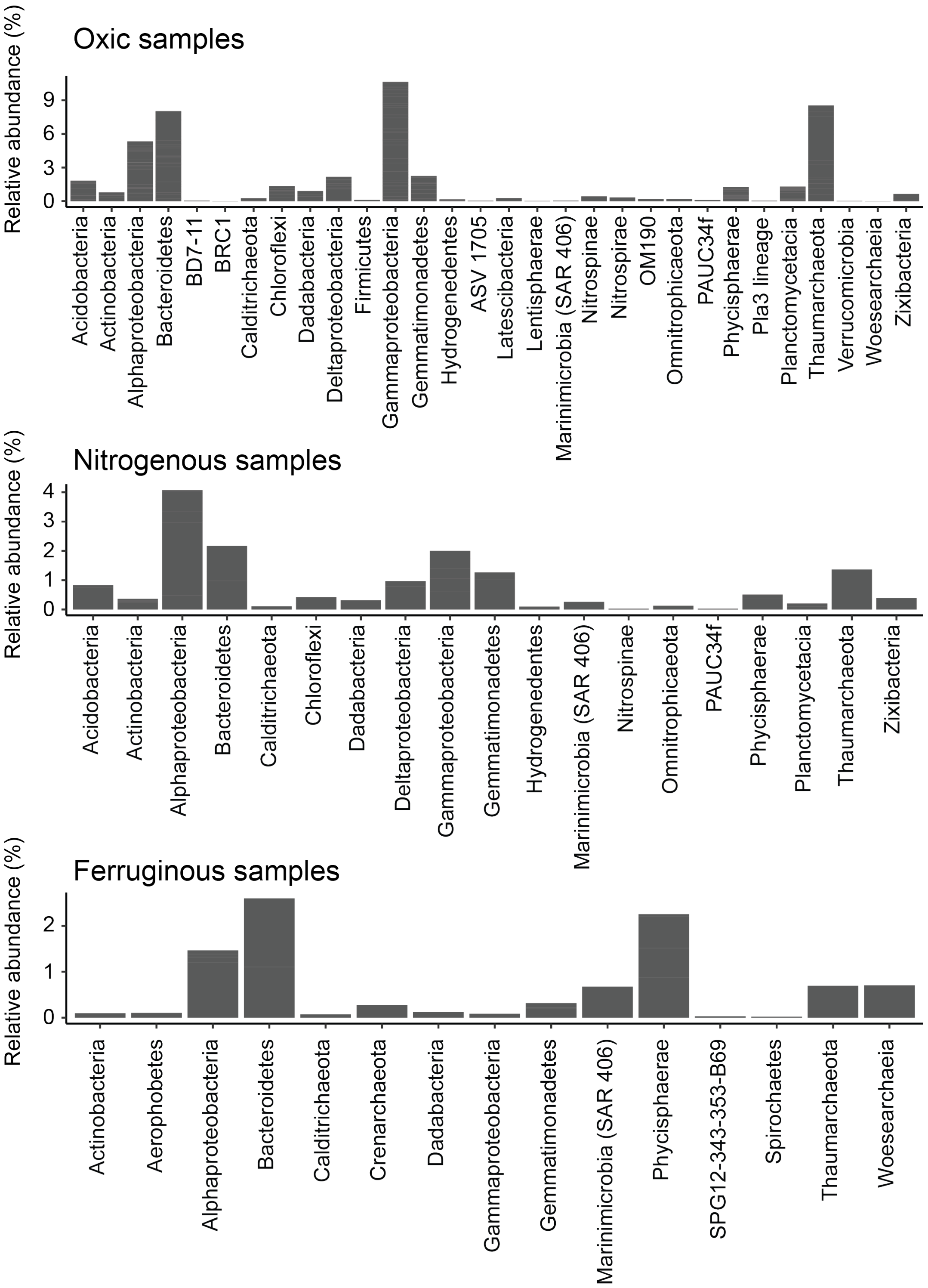

Supplement Figure 9:

(A) Number of ASVs and relative fractions of total reads constituted by the core-microbiomes of the oxic, nitrogenous, and ferruginous zones in the Atacama Trench. **(B)** Relative contributions of individual phyla/classes to the unique core-microbiomes of the oxic (yellow), nitrogenous (blue), and ferruginous zones (turquoise) and the ubiquitous core-microbiome ASVs in all redox zones (purple).

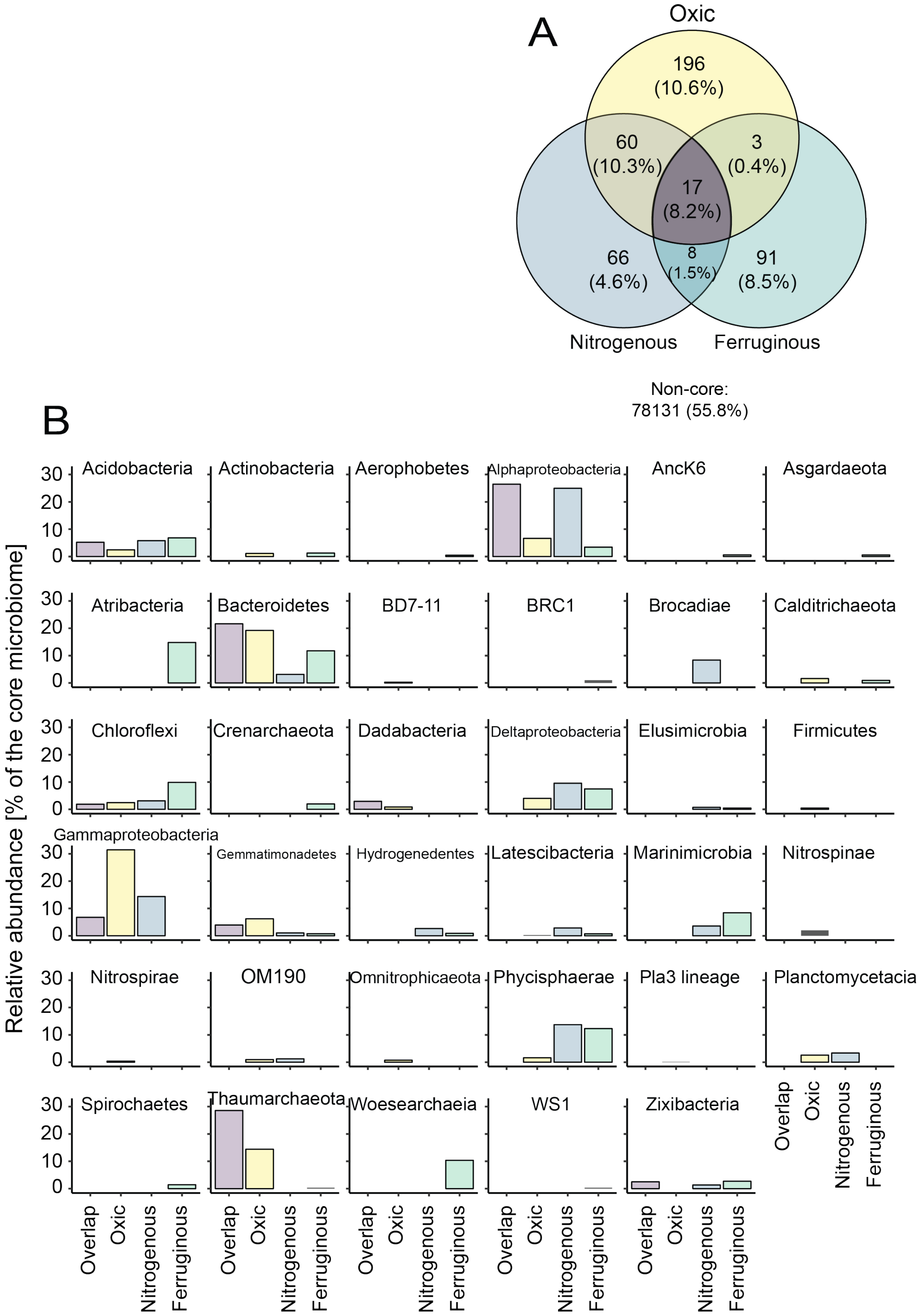

Supplement Figure 10:

Relative read abundance (%) of the 10 most abundant archaeal phyla in the archaea-specific 16S rRNA gene dataset grouped by redox zonation and sediment depth of the HR **(A)** and CR **(B)** sectioned hadal samples in the Atacama Trench. **(C & D)** Site-specific changes of these phyla with sediment depth.

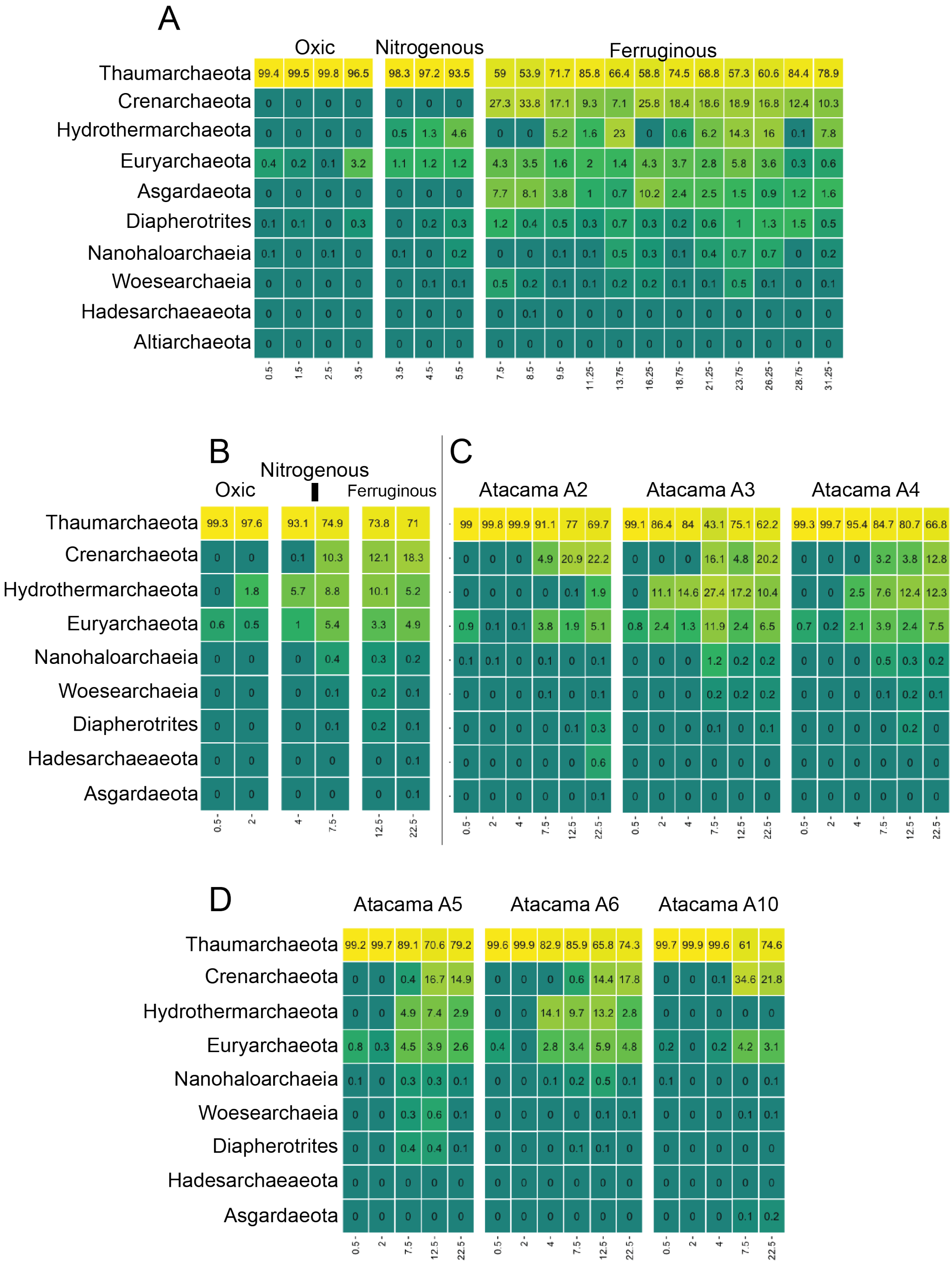

Supplement Figure 11:

Depth profiles of estimated absolute abundances of the 9 most abundant phyla/classes of Archaea in the hadal benthos based upon the universal 16S rRNA gene data. Colors correspond to the sampling site of each datapoint.

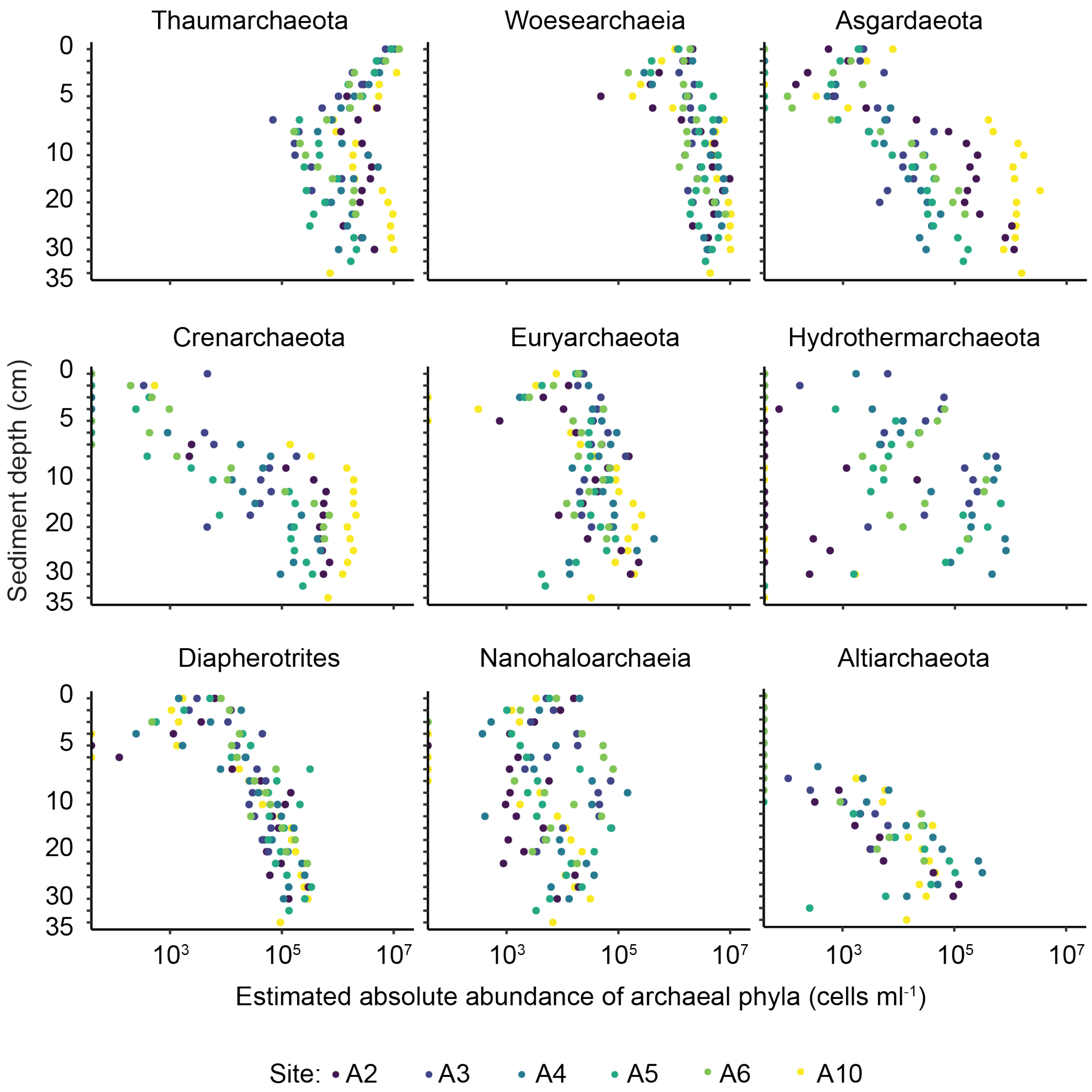

Supplement Figure 12:

Relative read abundance (%) of the 10 most abundant phyla/classes (grouped by sediment depth and site) based on the universal 16S rRNA gene **(A)** and archaea-specific 16S rRNA gene **(B)** data.

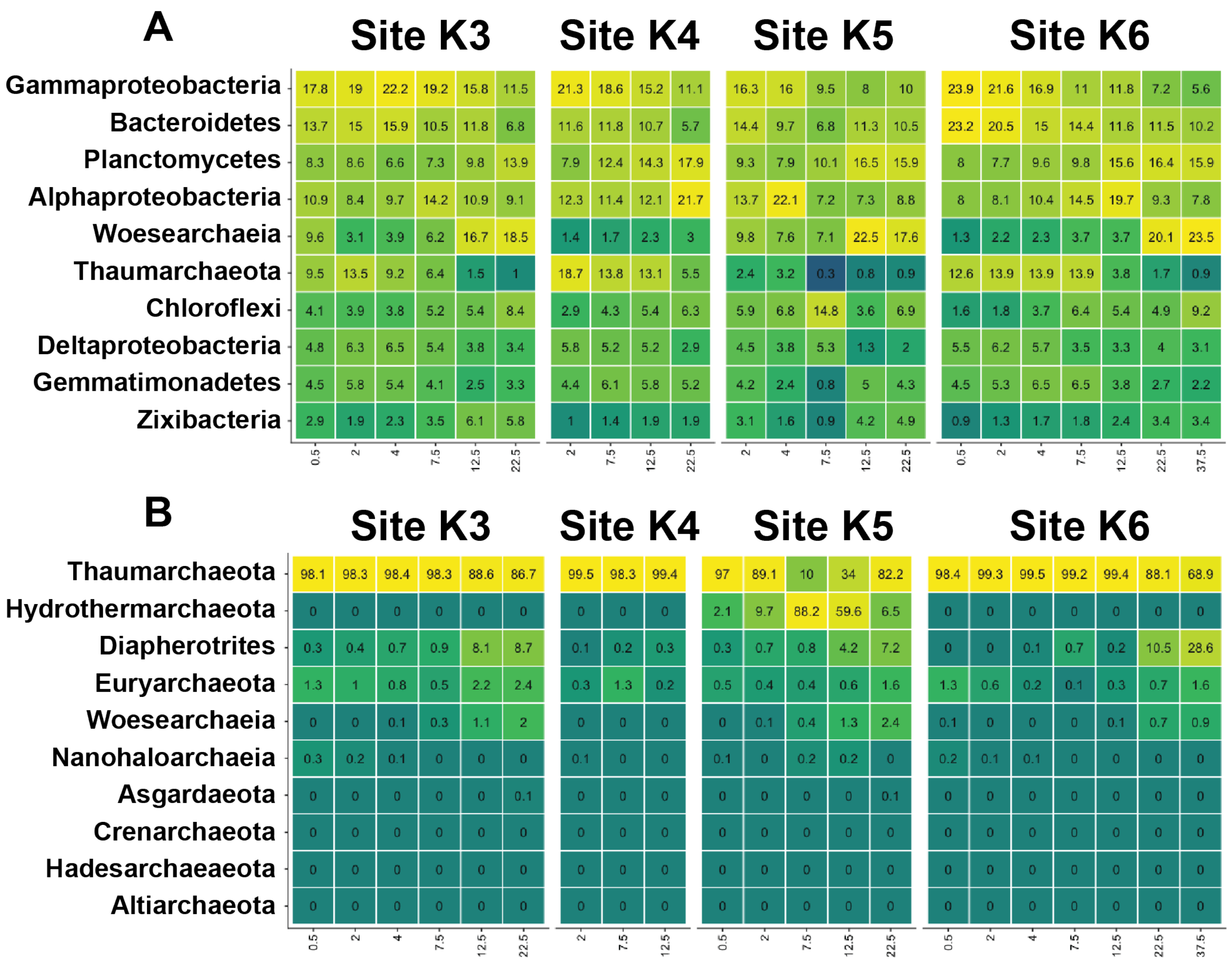

Supplement Figure 13:

Relative read abundance (%) on phylum/class level of the 10 most abundant lineages in the universal 16S rRNA gene **(A)** and archaea specific 16S rRNA gene **(B)** data grouped by sediment depth in the bathyal and abyssal sites of the Kermadec and Atacama trenches. **(C)** Estimated absolute abundances (cells mL^{-1}) of the 10 most abundant phyla/classes (universal 16S rRNA gene dataset) grouped by sediment depth in the bathyal and abyssal sites of the Kermadec and Atacama trenches.

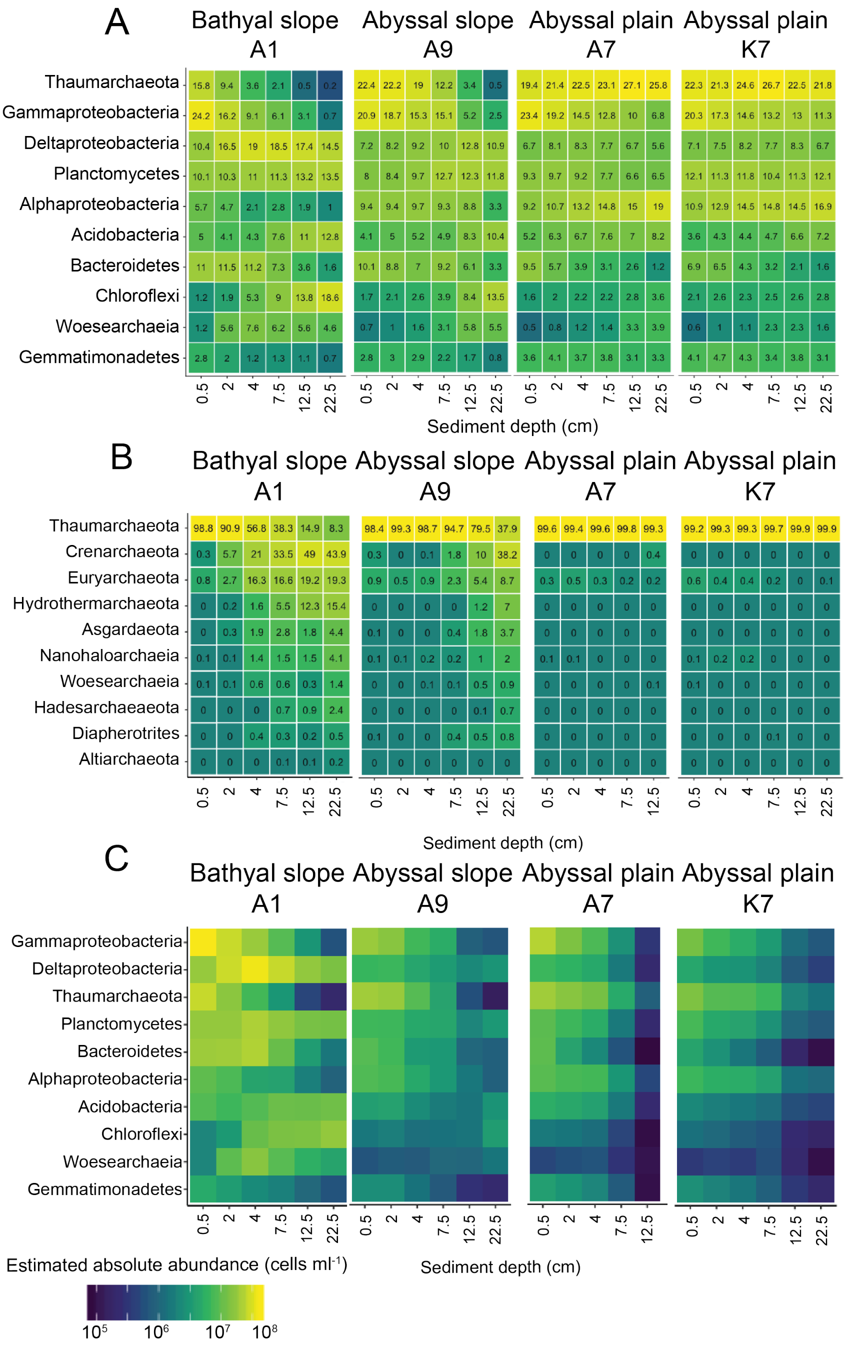

Supplement Figure 14:

Principle Coordinate Analysis (PCoA) of Bray Curtis dissimilarity across all CR samples **(A & B)** and all oxic samples **(C & D)** from the hadal (yellow), abyssal (purple) and bathyal (turquoise) realms in the Kermadec Trench (triangles) and Atacama Trench (circles) based upon the universal 16S rRNA gene **(A & C)** and archaea specific 16S rRNA gene **(B & D)** data.

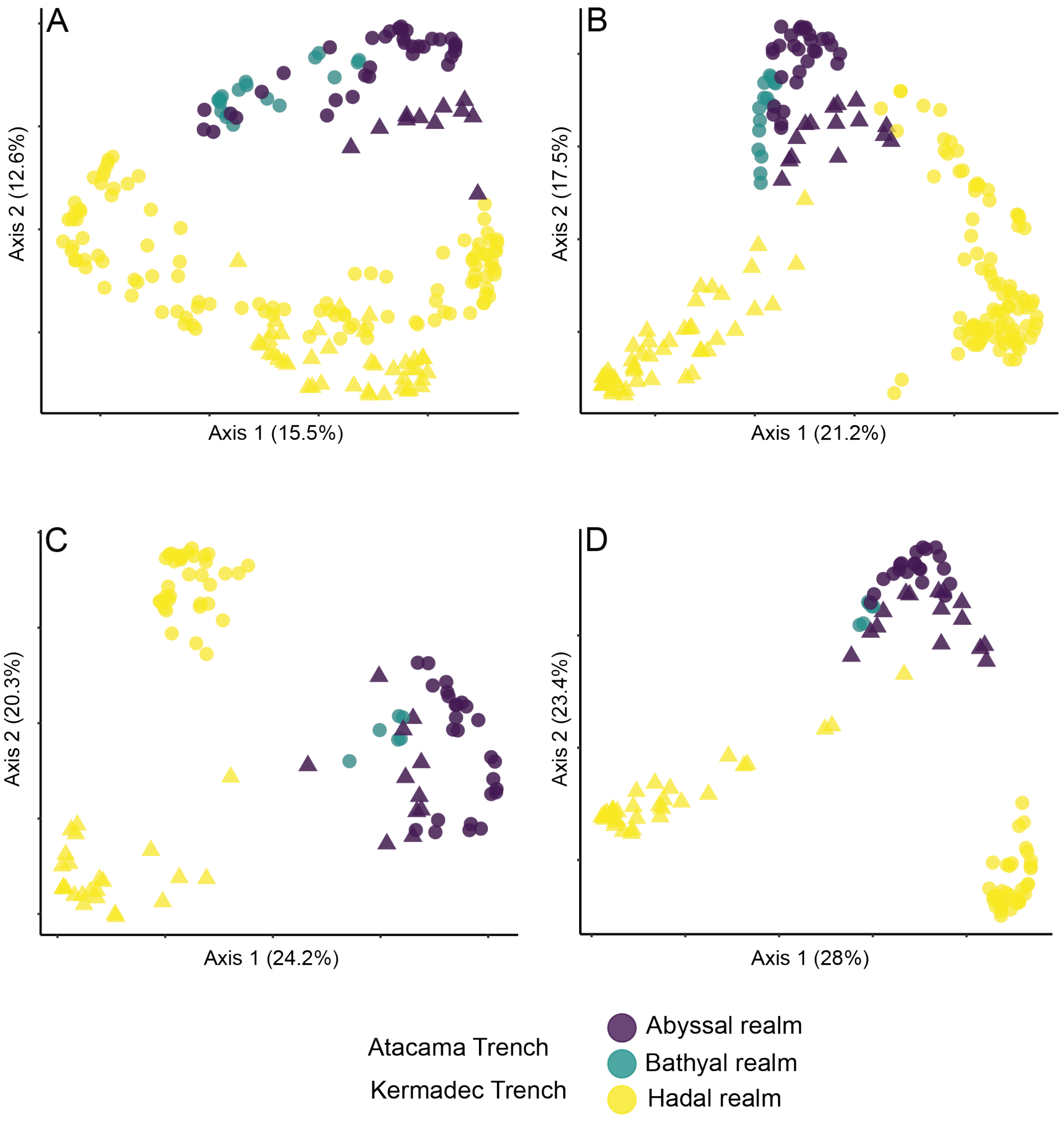

Supplement Figure 15:

(A) Number of ASVs and relative fractions of total reads constituted by the core-microbiomes of the oxic zones of the hadal, abyssal, and bathyal realms. **(B)** Relative read contributions of individual phyla/classes to the overlapping core-microbiomes of the oxic zones between the hadal-abyssal (purple), hadal-bathyal (turquoise), and abyssal-bathyal (yellow) and all realms (white).

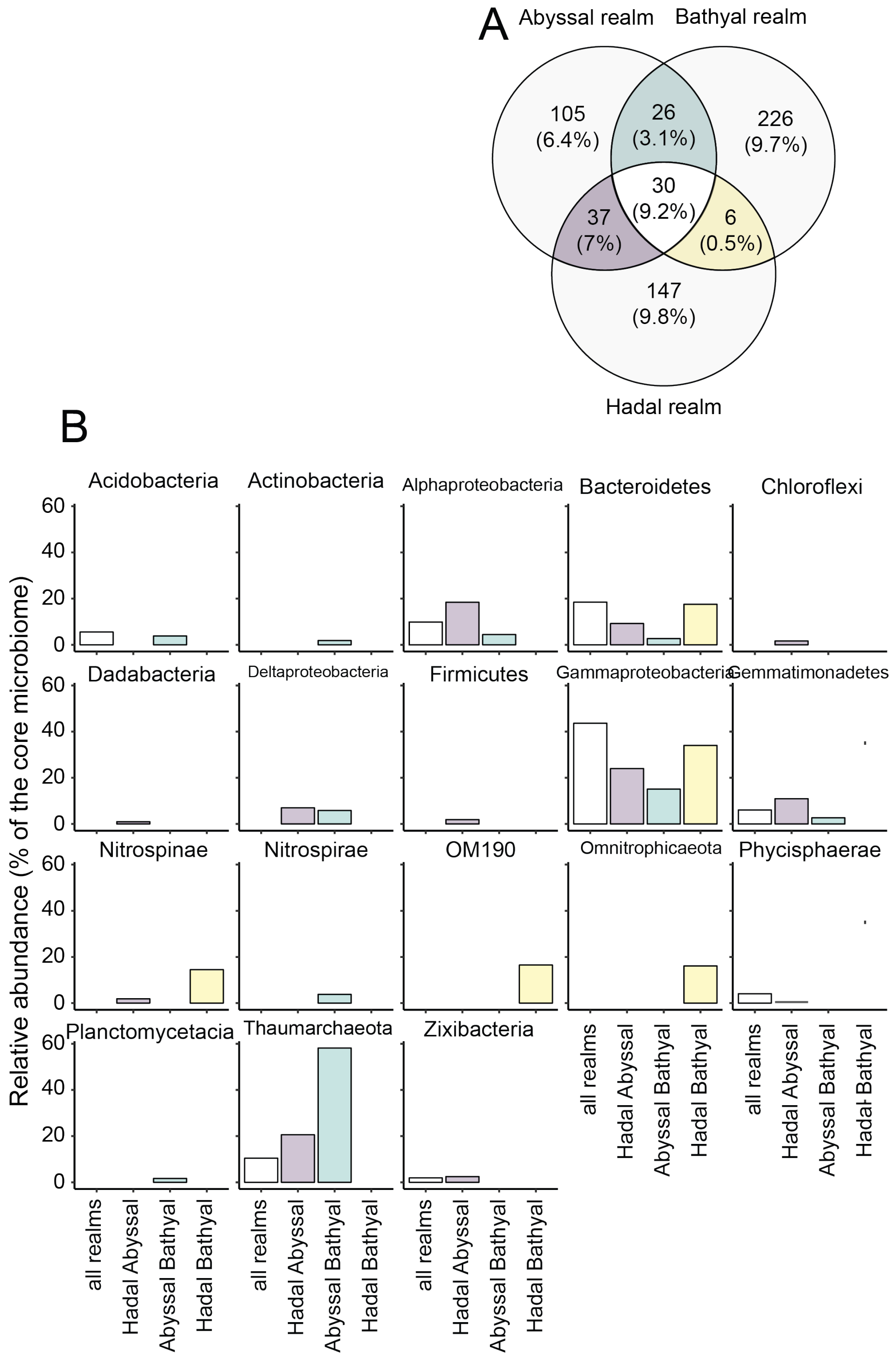

Supplement Figure 16:

Variation partitioning (%) of Hellinger transformed ASV counts between explanatory factors from hadal samples of the Atacama Trench **(A)**, across hadal samples from the Atacama and Kermadec trenches **(B)** and between oxic samples of all oceanic realms associated in the two trench systems **(C)**.

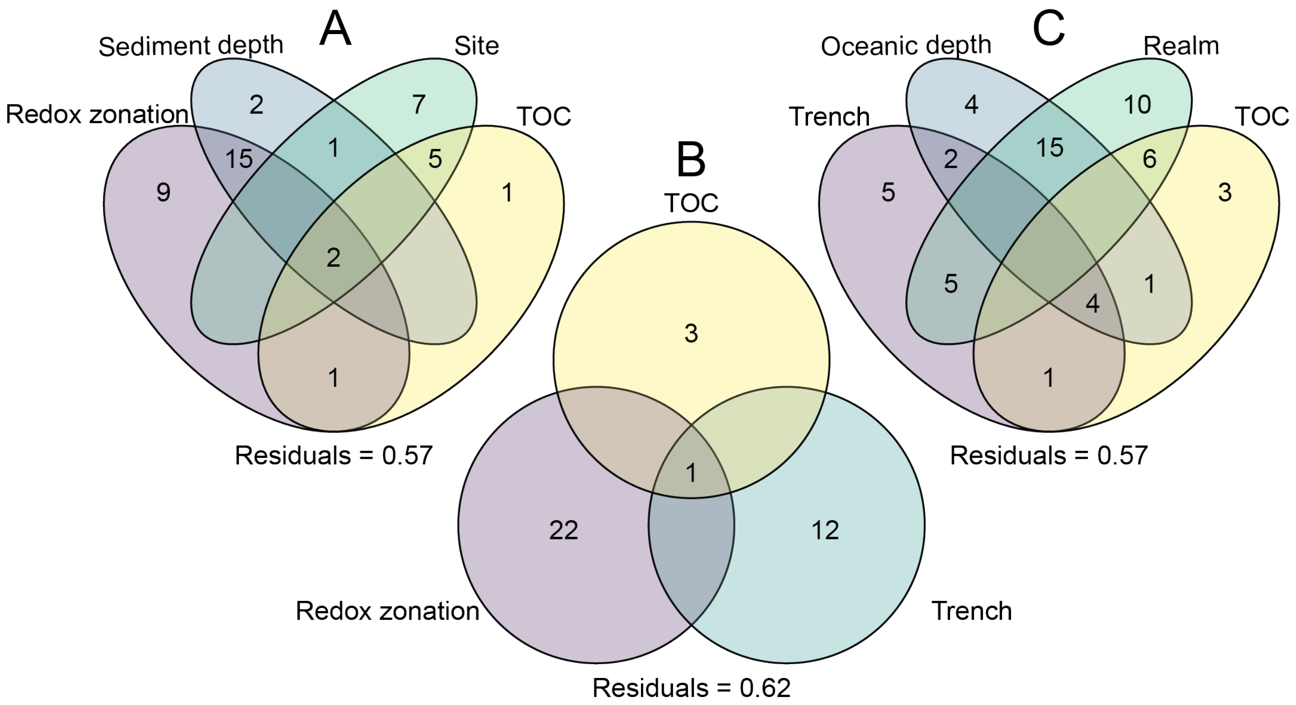

Supplement Figure 17:

Principle Coordinate Analysis (PCoA) of Bray Curtis dissimilarities between hadal samples from the Kermadec Trench (triangles) and Atacama Trench (circles) based on the universal 16S rRNA gene **(A)** and archaea-specific 16S rRNA gene **(B)** data. The color gradient describes sediment depth. **(C)** Redundancy analysis, using TOC, redox zonation and trench-trench factor as constraining variables. The data was initially transformed by applying the Hellinger transformation (Legendre & Gallagher, 2001). The relative contributions (eigenvalue) of each axis to the total inertia in the data, as well as to the constrained space only, are indicated in percent by the axis titles.

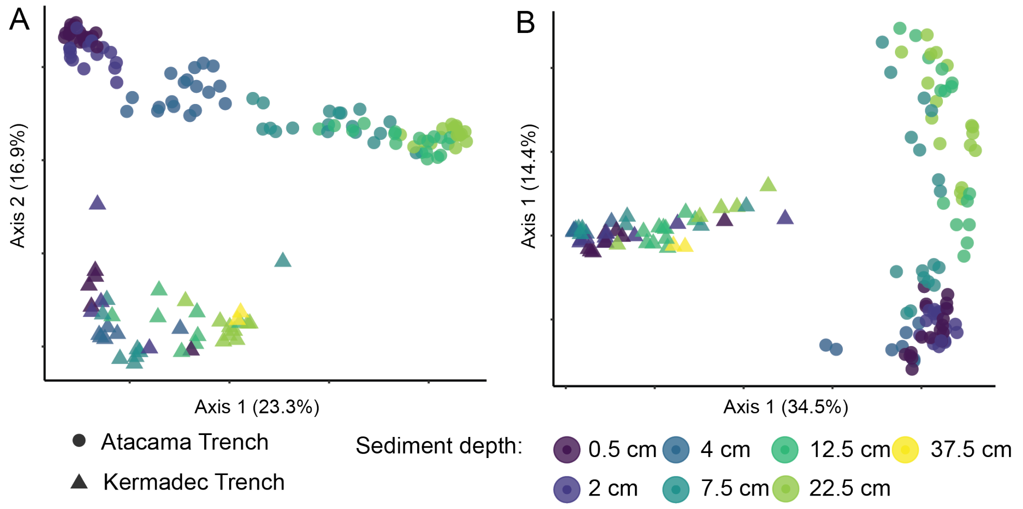

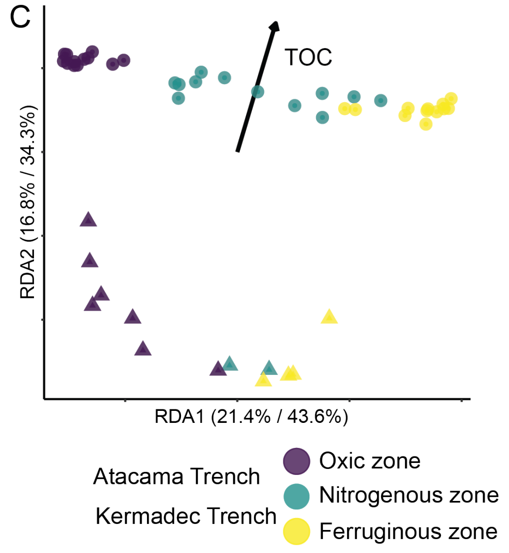

Supplement: Supplementary file 1 — Supplementary information [file 41396_2021_1021_MOESM1_ESM.pdf]
